# Direct Coupling of Methane and Carbon Dioxide on Tantalum Cluster Cations

**DOI:** 10.1002/chem.202203259

**Published:** 2022-12-27

**Authors:** Jozef Lengyel, Nikita Levin, Milan Ončák, Konstantin Jakob, Martin Tschurl, Ueli Heiz

**Affiliations:** ^1^ Lehrstuhl für Physikalische Chemie TUM School of Natural Sciences Technische Universität München Lichtenbergstraße 4 85748 Garching Germany; ^2^ Institut für Ionenphysik und Angewandte Physik Universität Innsbruck Technikerstr. 25 A-6020 Innsbruck Austria; ^3^ Lehrstuhl für Theoretische Chemie TUM School of Natural Sciences Technische Universität München Lichtenbergstraße 4 85748 Garching Germany

**Keywords:** bond activation, CO_2_ conversion, gas-phase catalysis, methane activation, tantalum cluster

## Abstract

Understanding molecular‐scale reaction mechanisms is crucial for the design of modern catalysts with industrial prospect. Through joint experimental and computational studies, we investigate the direct coupling reaction of CH_4_ and CO_2_, two abundant greenhouse gases, mediated by Ta_1,4_^+^ ions to form larger oxygenated hydrocarbons. Coherent with proposed elementary steps, we expose products of CH_4_ dehydrogenation [Ta_1,4_CH_2_]^+^ to CO_2_ in a ring electrode ion trap. Product analysis and reaction kinetics indicate a predisposition of the tetramers for C−O coupling with a conversion to products of CH_2_O, whereas atomic cations enable C−C coupling yielding CH_2_CO. Selected experimental findings are supported by thermodynamic computations, connecting structure, electronic properties, and catalyst function. Moreover, the study of bare Ta_1,4_^+^ compounds indicates that methane dehydrogenation is a significant initial step in the direct coupling reaction, enabling new, yet unknown reaction pathways.

## Graphical abstract

**How the tantalum cluster cations catalyze**: The cationic tantalum atom and the cationic tetramer exhibit a strong size‐dependent reactivity with methane and carbon dioxide: The monomer yields products from C−C coupling reactions, while the tetramer enables the formation of formaldehyde or syngas.

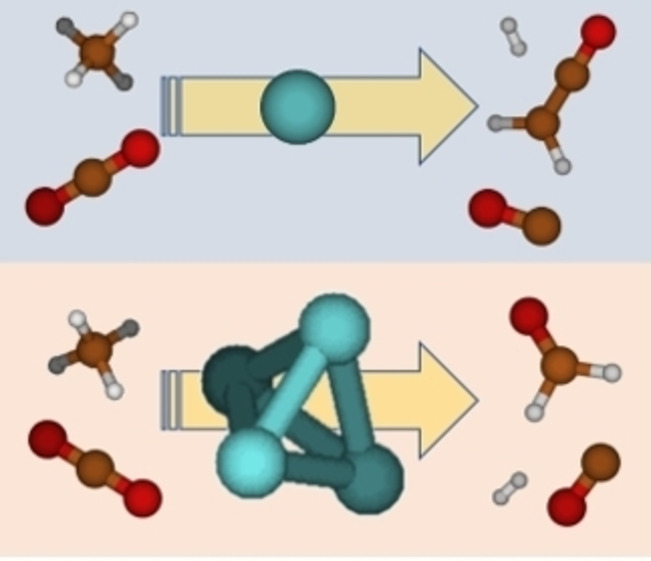

## Introduction

Direct conversion of carbon dioxide (CO_2_) and methane (CH_4_) producing liquid fuels and valuable feedstocks, for example, methanol, provides an excellent strategy for efficiently utilizing these greenhouse gases in a single step. When facing challenges such as climate change and a human's reliance on fossil fuels, the design and application of heterogeneous catalysts towards the activation of these small molecules is one of the main focuses of modern chemistry.^[1]^ Until now, only dry reforming of CH_4_ and CO_2_ has been applied on an industrial scale to produce syngas (CO+H_2_).^[2]^ Another, very attractive route is the catalytic coupling of CH_4_ and CO_2_ under mild conditions, yielding higher oxygenated hydrocarbons. However, it requires a catalyst enabling simultaneous CH_4_ and CO_2_ activation followed by the coupling of the two substrates, i. e., C−C or C−O bond formations.^[2b]^ Although there are a few reports of such processes in heterogeneous catalysis,^[3]^ they usually exhibit relatively poor selectivity and low yields of the desired products like acetic acid. This makes them commercially not yet very competitive.

Developing a powerful heterogeneous catalyst for this reaction attracts considerable attention in research. A complete understanding of the involved reaction mechanisms and their relation to the catalyst's material properties is, however, still lacking in many cases; this is particularly true for commercially applied systems.^[4]^ This predicament has different causes, like, for example, material complexity (structural, electronic, etc.) of the system or just the discrepancies between *in operando* reaction conditions and those during characterization of the catalyst.^[4b]^ In this regard, it is often useful to consider a highly defined model system in order to gain insight into the function of relevant catalysts. For instance, isolated atoms or clusters, which mimic active sites of heterogeneous catalysts, may be studied in the gas phase in order to elucidate fundamental reaction mechanisms on an atomic scale.^[5]^ While a fair number of studies focuses on either activation of methane or carbon dioxide, the number of papers discussing the direct coupling reaction of CO_2_ and CH_4_ can be counted on one hand.^[6]^ The choice of the elemental composition of the active site is highly limited as both activation and cleavage of C−H bonds in methane and the reduction of carbon dioxide need to be promoted by the catalyst. Although a considerable number of third‐row transition metal cations probed as single‐atom catalysts meet the first criterion, only W^+^ and Ta^+^ also show the exothermic activation of carbon dioxide.^[7]^ Particularly interesting is the coupling of methane and carbon dioxide mediated by atomic tantalum cations.^[6a]^ In this process, methane is first activated on isolated Ta^+^ to yield a metal‐carbene complex TaCH_2_^+^, which is further functionalized by CO_2_ insertion forming C_2_H_2_O, most likely with ketene structure.^[6a]^ By now, only two more species, namely CuB^+^ and RhVO_3_^−^, have been reported as viable catalysts for the coupling reaction.[^6b^, ^6c^] Tantalum as a potential catalyst has proven to be versatile and can be used in a large variety of coupling reactions as recently demonstrated on the non‐oxidative coupling of methane,^[8]^ methane oxidation,^[9]^ and ammonia synthesis.^[10]^

Herein, a combined approach of experiment and computation is chosen to unravel the reaction mechanism of the coupling of CO_2_ and CH_4_ mediated by tantalum cations of varying sizes in light of recent observations in our group, described in the following. First, it was shown that the reactivity of Ta_*n*_^+^ ions with *n*=1–5 heavily depends on the cluster size *n*, exhibiting strong size effects.^[11]^ For example, Ta_5_^+^ was found to be inert with respect to methane dehydrogenation, while Ta_4_^+^ reacts with methane yielding a [Ta_4_CH_2_]^+^ species. Second, spectroscopic experiments of these intermediates revealed significant discrepancies between atomic and tetrameric tantalum cations: The former favors the formation of carbene structures, while in case of the latter carbyne hydrides and carbide dihydrides are formed with high selectivity.^[12]^ Therefore, we mainly focus on the various pathways of the CO_2_ activation by the products from methane dehydrogenation mediated either by single or tetrameric tantalum cations, in order to elucidate the role of different [Ta_1,4_CH_2_]^+ ^isomers in the coupling reaction.

## Results and Discussion

### Reaction of the size‐selected [Ta_n_CH_2_]^+^(n=1,4) complexes with CO_2_

**Figure 1:**
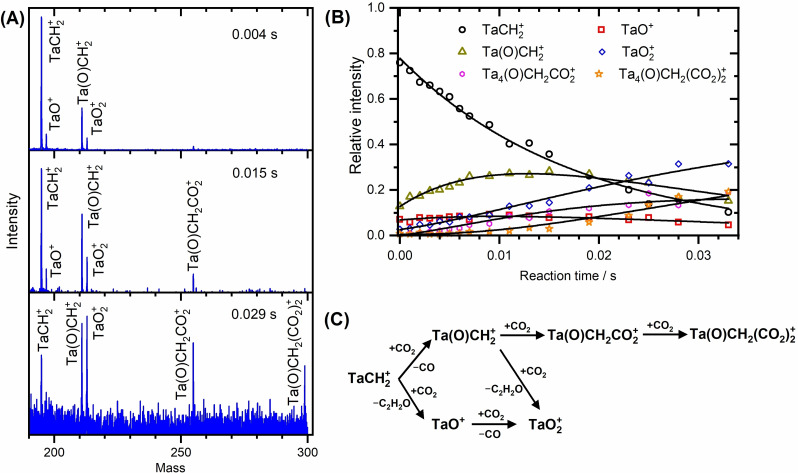
A) Mass spectra taken at different reaction times and B) kinetic analysis of the reaction of mass‐selected TaCH_2_^+^ ions with CO_2_. The reactions are conducted at room temperature under multi‐collisional conditions in the presence of He buffer gas with 100 ppm CO_2_ at a total pressure of 0.82 Pa. C) Panel represents the underlying mechanism suggested by the kinetic fit.

As several studies reported the dissociative adsorption of methane to a metal core to be the first step in the CH_4_/CO_2_ coupling,^[13]^ we focus in our first experiment on the reaction of the size‐selected [TaCH_2_]^+^ complexes with carbon dioxide, i. e., Ta^+^ species that already reacted with methane. By activating methane prior to the reaction, we also significantly simplify the reaction mechanism by eliminating collisions of reaction intermediates with additional methane molecules. Mass spectra, shown in Figure 1, reveal a total of six different species in the reaction sequence, namely TaCH_2_^+^, TaO_1,2_^+^, Ta(O)CH_2_^+^, and Ta(O)CH_2_(CO_2_)_1,2_^+^. The underlying reaction mechanism determined by pseudo‐first order kinetics confirms the process to start with two competing parallel reactions. While Ta(O)CH_2_^+^ is formed through CO_2_ deoxygenation on the carbene, the presence of TaO^+^ indicates that TaCH_2_^+^ can also undergo a C−C coupling with CO_2_ followed by the loss of a C_2_H_2_O moiety. Both intermediates can, however, react further with CO_2_: TaO^+^ can deoxygenate another molecule of CO_2_ to form TaO_2_^+^, and Ta(O)CH_2_^+^ is converted either into Ta(O)CH_2_(CO_2_)_1,2_^+^ through adsorption of CO_2_ or into TaO_2_^+^ by virtue of a possible C−C coupling with concomitant elimination of a C_2_H_2_O moiety. Overall, the primary gaseous reaction products are postulated to be CO formed in a deoxygenation step and ketene, CH_2_CO (ethenone). Note that the formation of the ketene is energetically favored over other possible structural isomers such as ethynol or an oxirene.^[6a]^

It should be stressed that the observation of the two‐step coupling, i. e., deoxygenation followed by coupling, is in good agreement with the earlier measurement of Wesendrup and Schwarz performed under single collisional conditions.^[6a]^ Our rate coefficient for coupling (4.2×10^−9^ cm^3^ s^−1^; see Table S1) is, however, significantly higher than in the FT‐ICR experiment (1.3×10^−10^ cm^3^ s^−1^) reported in that study, related most likely to the different reaction conditions in both studies. In addition to that, the kinetic fit identifies a new coupling reaction, which occurs directly from TaCH_2_^+^ in a single‐step process without the necessity to generate Ta(O)CH_2_^+^. A more detailed kinetic analysis reveals that CO_2_ deoxygenation as a first step is preferred over C−C coupling with a branching ratio of 5 : 1. Yet, as the subsequent C−C coupling on Ta(O)CH_2_^+^ is suppressed by a factor of 2.2 in comparison to CO_2_ adsorption (3 : 7), the contribution of both pathways to the formation of CH_2_CO yields ∼40 %.

**Figure 2:**
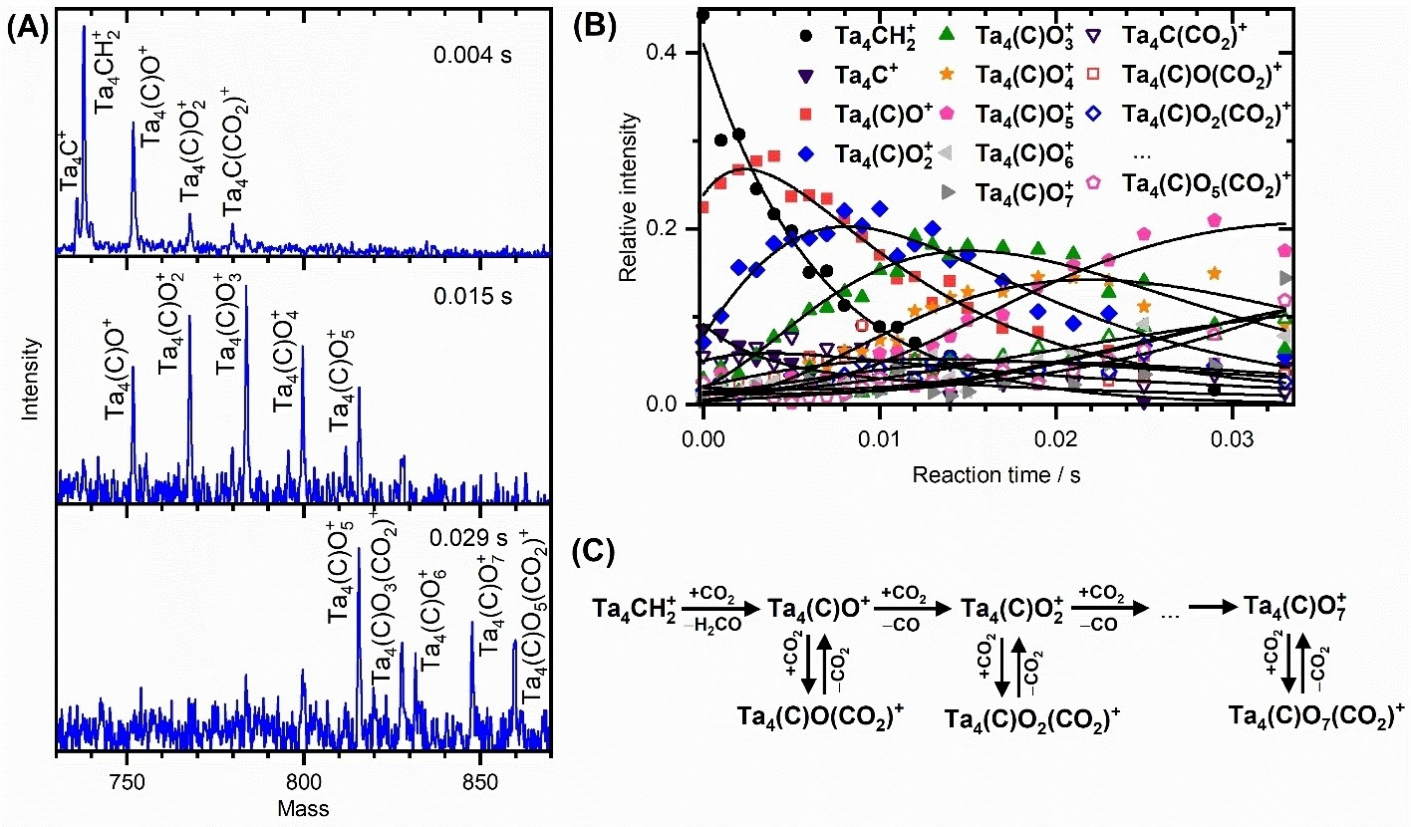
A) Mass spectra taken at different reaction times and B) kinetic analysis of the reaction of mass‐selected [Ta_4_CH_2_]^+^ ions with CO_2_. The reactions are conducted at room temperature under multi‐collisional conditions in the presence of He buffer gas with 100 ppm CO_2_ at a total pressure of 0.82 Pa. C) Panel represents the underlying mechanism suggested by the kinetic fit. Note that there are distinct symbols and colors associated with each species. The symbols for CO_2_‐adsorbed analogs are identical, except that they have no filling.

As the low ion abundance of bare Ta_2_^+^ and Ta_3_^+^ (see Figure S3) impedes the study of their products from methane dehydrogenation, the only remaining cluster that facilitates this reaction with this hydrocarbon is Ta_4_^+^.^[11b]^ In the reaction of the size‐selected [Ta_4_CH_2_]^+^ with CO_2_, several species are detected in the mass spectra, as shown in Figure 2. Within the first 4 ms, the mass spectrum is dominated by the reactant, as well as a less intense signal associated with Ta_4_(C)O^+^ and traces of Ta_4_C^+^, Ta_4_(C)O_2_^+^, and Ta_4_C(CO_2_)^+^. As the reaction progresses, further oxygenated species, Ta_4_(C)O_2–7_^+^, appear, along with CO_2_ adsorption products, Ta_4_(C)O_1–7_(CO_2_)^+^. According to the kinetic models, Ta_4_CH_2_^+^ is exclusively dissociated either into a formaldehyde‐like (H_2_CO) species or a syngas (H_2_+CO) with an overall rate coefficient of 13.1×10^−9^ cm^3^ s^−1^ (see Table S2). After that, further carbon dioxide molecules are either adsorbed or deoxygenated. It should be noted that Ta_4_(C)O(CO_2_)^+^ ions are considerably lower in intensity than Ta_4_(C)O_2_^+^ and rapidly disappear with decreasing intensity of the corresponding reactant ion, i. e., Ta_4_(C)O^+^, as a result of a reversible reaction.

### DFT modeling of CO_2_ activation by [Ta_n_CH_2_]^+^ (n=1,4) ions

From the above results we conclude that there is a fundamental difference between single atoms and clusters in their ability to mediate CH_4_ and CO_2_ coupling. Whereas the single atom promotes C−C coupling, the tetramer facilitates C−O coupling. It ought to be mentioned that parts of the atomic tantalum ion pathway have been examined extensively in the literature.[^6a^, ^14^] However, as described above, a single step coupling was identified in this experiment, and therefore the respective quantum chemical exploration for the second reaction channel has not been discussed yet. To this aim, we used hybrid DFT calculations with HSE06 functional with a subsequent single‐point CCSD refinement to calculate ground state energies and relevant thermochemical properties of the suggested mechanism (see Supporting Information for details); the wave function was checked for stability prior to every calculation, all calculations were performed in Gaussian.^[15]^

The primary focus is to differentiate between the two channels leading to the same reaction outcome, TaO_2_^+^:

(1) $$TaCH_{2}^{+}+2\;\;CO_{2}\to TaO_{2}^{+}+CO+CH_{2}CO$$

(Ia) $$TaCH_{2}^{+}+CO_{2}\to Ta\left(O\right)CH_{2}^{+}+CO$$

(Ib) $$Ta\left(O\right)CH_{2}^{+}+CO_{2}\to TaO_{2}^{+}+CH_{2}CO$$

(IIa) $$TaCH_{2}^{+}+CO_{2}\to TaO^{+}+CH_{2}CO$$

(IIb) $$TaO^{+}+CO_{2}\to TaO_{2}^{+}+CO$$

**Figure 3:**
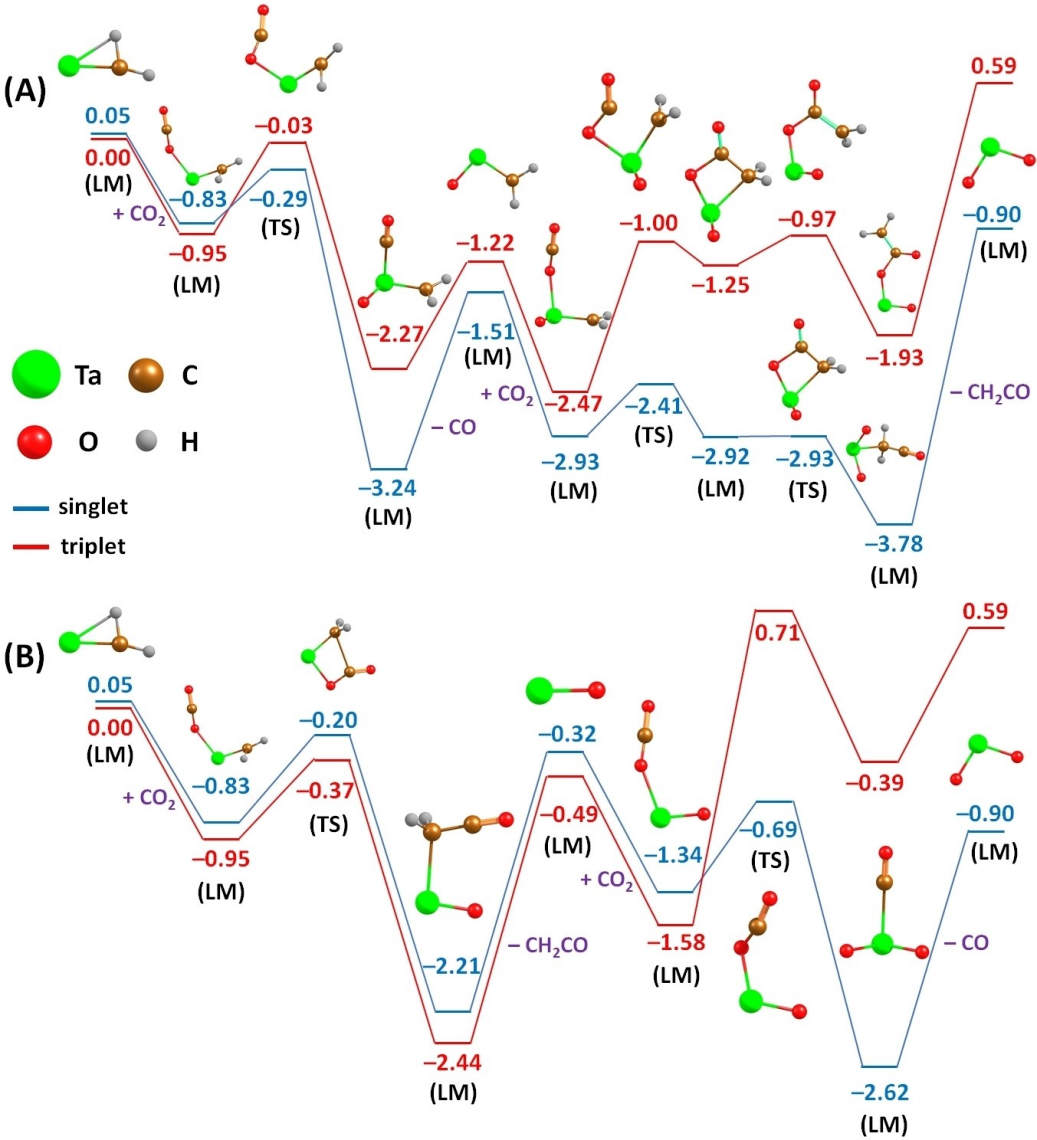
Reaction pathways calculated for coupling reactions mediated by a single tantalum ion, TaCH_2_^+^+CO_2_, A) channel I (following the calculations from Sändig and Koch^[14]^) and B) channel II. Calculation are performed at the CCSD/def2TZVP//HSE06/def2TZVP level of theory, with relative energies at 0 K given in eV inclusive of zero‐point energy corrections. The calculations in Panel A are in qualitative agreement with those of Sändig and Koch.^[14]^

The investigation of initial and final states reveals that the overall reaction is exergonic in the singlet case with a reaction energy of Δ*E*_r_=−0.94 eV (Δ*G*_r_ *°*=−1.02 eV; Figure 3A, blue curve) and endergonic in the triplet with Δ*E*_r_=0.59 eV (Δ*G*_r_ *°*=0.48 eV, red curve). Other multiplicities are not considered in the following investigation as these are energetically unfavorable.^[12a]^ This striking difference in reaction energy arises as the triplet state is slightly favored in the case of the carbene (by 0.05 eV) while TaO_2_^+^ strongly favors a singlet configuration (by 1.49 eV). Interestingly, the situation is opposite in the case of the involved reaction intermediates. While the carbene Ta(O)CH_2_^+^ prefers to be in the singlet state (0.29 eV), the energetic minimum for the tantalum monoxide, TaO^+^, is the triplet state (0.17 eV). Therefore, spin‐crossing effects are likely to occur in this process.^[16]^

Focusing on the first reaction channel, which has already been thoroughly investigated theoretically by Sändig and Koch,^[14]^ one finds that the overall formation of the reaction intermediate (i. e., Ta(O)CH_2_^+^) is energetically downhill in the cases of both multiplicities, even though the reaction is favored more strongly for singlet states, as shown in Figure 3(A). The consecutive conversion of this intermediate with another CO_2_ into the final state is endergonic for both spin multiplicities, but the calculated barriers lie below the energy of the exit channel as only the desorption of the ketene requires additional energy. At the same time, the adsorption of carbon dioxide is exergonic with an energy of 1.2–1.4 eV, meaning that adsorption of CO_2_ is energetically strongly preferential in this case. This is in good agreement with the experimental observations for tantalum carbene cations, as Ta(O)CH_2_^+^ preferentially undergoes CO_2_ adsorption over ketene formation when reacting with CO_2_ (see Table S1). In the second reaction channel (Figure 3B), the formation of TaO^+^ and CH_2_CO is considerably less exergonic, showing path II to be less favorable than path I. However, due to a strong chemisorption of the evolving ketene, the reaction is likely to occur, regardless of the overall reaction energy. In this case, the TaCH_2_^+^ acts like a typical nucleophilic Schrock carbene, which explains its tendency to allow direct C−C coupling using carbon dioxide; here, the carbon atom in CO_2_ is positively polarized. The following product formation, TaO_2_^+^, is then significantly exergonic for singlet states, in contrast to reaction path I, and strongly endergonic for triplet states. Hence, the triplet state of TaO^+^ can be considered as a trap state, as its formation is energetically favorable, while consequent reactions are energetically unlikely. Overall, the thermochemical calculations are in line with the observed experimental branching ratios. The calculations correctly predict that CO_2_ deoxygenation, leading to formation of Ta(O)CH_2_^+^, is favored over the one‐step coupling (5 : 1 in the experiment). Further, the initial CO loss in path I might proceed directly from the CO_2_TaCH_2_^+^ isomer, bypassing the need to go over the high‐lying transition state at −0.29 eV in triplet multiplicity. Also, the reaction intermediate Ta(O)CH_2_^+^ energetically prefers CO_2_ adsorption over C−C coupling, in coherence with the experimental branching ratio of 7 : 3. However, as the calculations predict this branching to be too much in favor of CO_2_ adsorption, it is important to mention that this adsorption process might also be a crucial part of the coupling reaction. This enables the formation of the transition state, along with a gradual thermalization of the ion under experimental conditions, which leads to the stabilization of the complex with adsorbed CO_2_.

**Figure 4:**
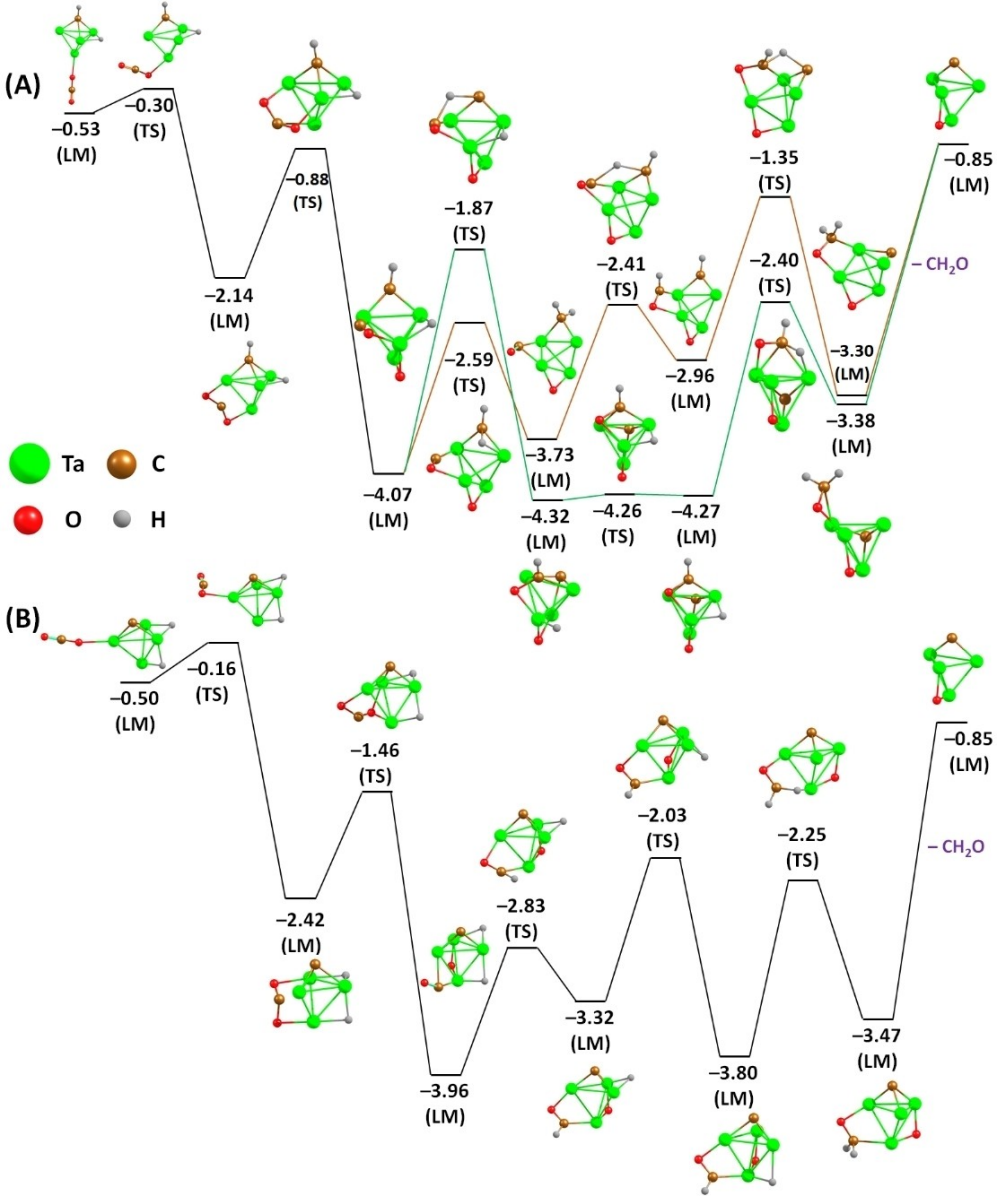
Selected structures along the [Ta_4_CH_2_]^+^+CO_2_ reaction pathway in doublet multiplicity with A) carbyne hydride and B) carbide dihydride. Relative energies at 0 K, calculated at the CCSD/def2TZVP//HSE06/def2TZVP level of theory, are given in eV inclusive of zero‐point energy corrections and with respect to HTa_4_CH^+^+CO_2_ (see Figures S5 and S6 for the complete pathways).

While for atomic tantalum ions the spin state plays a fairly significant role, it is known that cationic tantalum tetramers prefer to be in a doublet state.^[11a]^ Since the higher number of atoms leads to an increase in structural complexity, several structural isomers of the involved species must be considered. In our previous study,^[12c]^ structures and energetics of the possible initial structural isomers have already been discussed in detail. It was found that carbyne hydride (HTa_4_CH^+^) and carbide dihydride (H_2_Ta_4_C^+^) isomers are favored over the carbene one. On the tantalum tetramers, the single charge can distribute somewhat evenly among the four different atoms, which consequently leads to a decrease in partial charge. This destabilizes the Schrock carbene compared to the other structural isomers, as the tantalum sites are less electrophilic. The investigation of the final state also examines possible formation of two different structural isomers of Ta_4_(C)O^+^: a carbide oxide (CTa_4_O^+^) and a carbonyl (Ta_4_CO^+^). Structural optimization, however, reveals a striking energy difference of more than 3.0 eV, strongly favoring CTa_4_O^+^. Hence, from a thermodynamic point of view the formation of a carbide oxide during the above reaction is preferred. Our reaction pathway calculations (Figure 4) also show that all barriers indeed lie below the energy of the entrance channel, although the formation of a carbide oxide requires the cleavage of two C=O bonds, while formation of a carbonyl might only require one to be broken. Similarly, one also has to consider the structure of the dissociated CH_2_O species leaving the Ta_4_CH_2_(CO_2_)^+^ cluster, which can be either a formaldehyde molecule (H_2_CO) or a mixture of CO and H_2_ (also known as syngas). In terms of entropy, the syngas formation seems to be preferable, since coupling reduces the number of molecules and each molecule in the gas phase has a high translation entropy. In contrast, the formation of H_2_CO is energetically favorable over the formation of syngas by 0.13 eV.^[17]^ This difference is relatively small compared to the large difference between the different tantalum structural isomers. However, a strong chemisorption of formaldehyde on both tantalum species considered indicates the formaldehyde likely to be formed. Accordingly, the calculated reaction pathways focus exclusively on H_2_CO formation. Further, our quantum chemical calculations also show that hydrogen evolution directly from H_2_Ta_4_C^+^ would be connected to a reaction barrier of Δ*E*_ZPE_=1.39 eV (see Figure S4) by considering zero‐point energies and is hence unlikely. While the presence of a signal corresponding to Ta_4_C^+^ may be interpreted as direct experimental evidence for exactly this process, the behavior of the relative intensity of this mass over time indicates this species to originate instead from an insufficient mass selection due to the small mass difference. The release of two hydrogens observed in the reaction of [Ta_4_CH_2_]^+^ with O_2_,^[9]^ may also be indicative for the formation of syngas rather than formaldehyde. However, the oxidation with O_2_ is a process of much higher exothermicity (as for example evidenced by pathways resulting in the fragmentation of the cluster) and the pathway mentioned in that work is only a minor reaction channel. In any case, the formation of syngas molecules represents a plausible option in addition to the release of formaldehyde, as such a reaction outcome has recently been reported for rhodium–titanium oxide anions.^[18]^ As a result, the CH_2_O species might be either a formaldehyde molecule, CO/H_2_, or a mixture thereof. Figure 4 also shows that a multitude of reaction pathways for CH_2_O formation has to be expected. The overall reaction equation can therefore be written as:

(2) $$\left[\mathrm{Ta}{}_{4}\mathrm{CH}{}_{2}\right]{}^{+}+\mathrm{CO}{}_{2}\to \mathrm{Ta}{}_{4}\left(C\right)O{}^{+}+H{}_{2}\mathrm{CO}\;\left(\mathrm{and}/\mathrm{or}\;H{}_{2}+\mathrm{CO}\right)$$

For both species, the atomic cation and the cationic tetramer of tantalum, the resulting products are energetically favored by less than 1 eV. Thus, they do not represent deep thermodynamic sinks and may be accessible for consecutive reactions leading to the closure of a catalytic cycle.

### Coupling of CH_4_ and CO_2_ on bare Ta_n_^+^ (n=1,4) clusters

**Figure 5:**
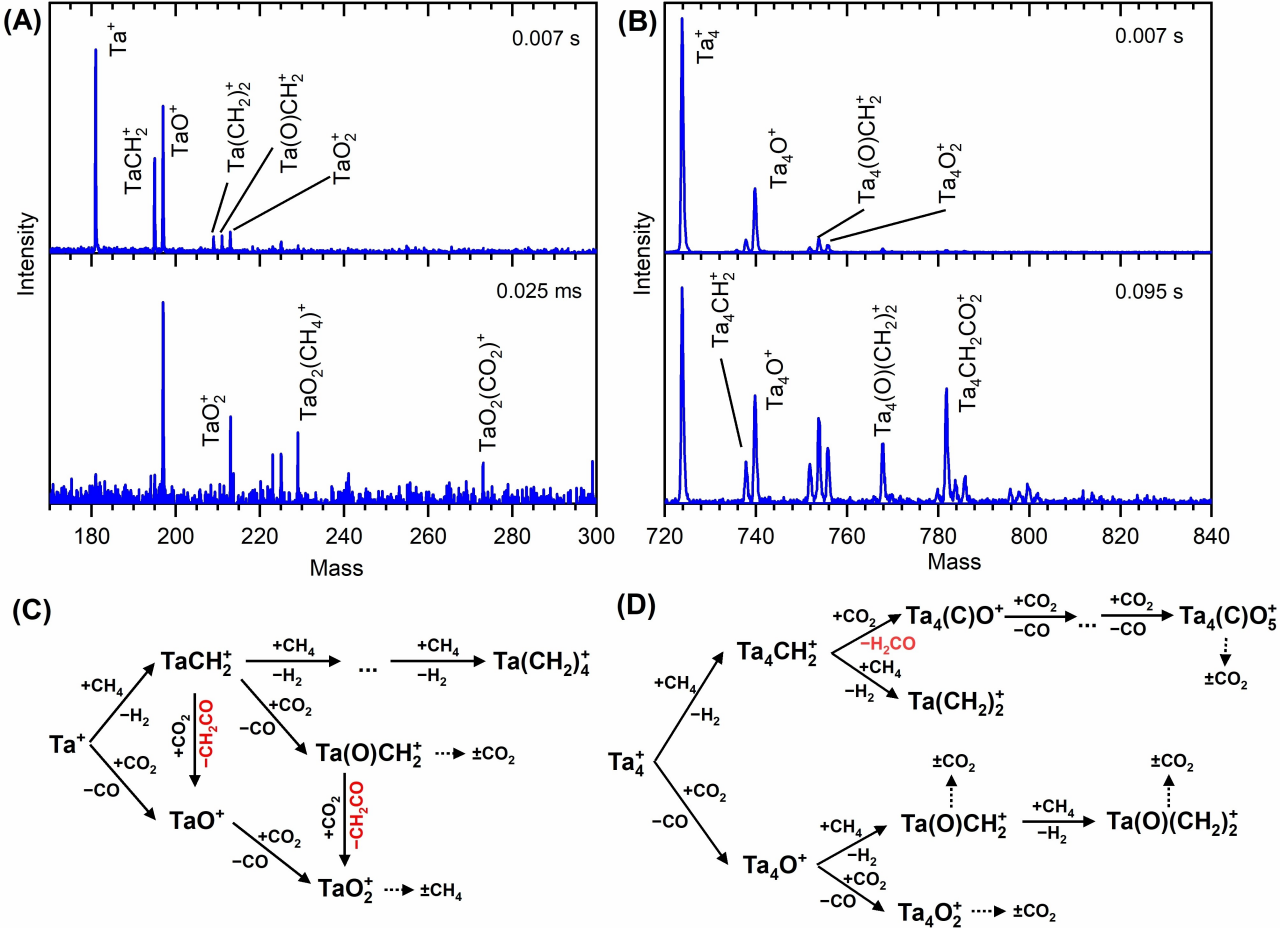
Mass spectra and mechanism of the reaction of bare Ta^+^ and Ta_4_^+^ ions with a mixture of CH_4_ and CO_2_. The reactions are conducted at room temperature under multi‐collisional conditions in the presence of He buffer gas with A) 20 ppm CO_2_ and 100 ppm CH_4_ and B) 25 ppm CO_2_ and 2500 ppm CH_4_ at a total pressure of 0.82 Pa. Panel C and D) represents the underlying mechanism for the reaction of Ta^+^ and Ta_4_^+^, respectively.

Finally, the capability of small clusters to mediate the coupling of methane and carbon dioxide is addressed experimentally by probing the respective reaction mediated by bare metal cluster ions. The bare, mass‐selected cluster cations created in the laser vaporization source are stored in the ion trap reactor, which is this time filled with a mixture of methane and carbon dioxide. Figure 5(C) illustrates the underlying sequence for the reaction of mass‐selected Ta^+^ cations with methane and carbon dioxide at 300 K under multi‐collisional conditions, for which the kinetic fits, shown in Figure S1, result in the corresponding rate coefficients given in Table S3. As the CO_2_ deoxygenation proceeds far more rapidly than the CH_4_ dehydrogenation, this reaction is probed by using a reaction gas mixture containing only small amounts of carbon dioxide with the concentration of methane in the buffer gas being one or two orders of magnitude greater. Despite the complex reaction scheme, four types of reactions are identified, namely CH_4_/CO_2_ coupling, CO_2_ deoxygenation, CH_4_ dehydrogenation, and adsorption of both precursors. The experiment shows that both coupling reactions occur as identified previously in this work for the reaction of the carbene with CO_2_: while one reaction takes place via the Ta(O)CH_2_^+^ intermediate, the other one results from the direct conversion of TaCH_2_^+^ with CO_2_. The latter reaction yields TaO^+^, which is also formed from the Ta^+^ oxygenation by CO_2_. However, kinetic modeling reveals that the dominant process is coupling with a rate of (2.0±0.7)×10^−8^ cm^3^ s^−1^ while decarbonylation proceeds more than three times slower (see Table S3). It must be stressed that only the inclusion of both coupling reactions in the reaction sequence is capable to replicate the experimental data. The other reactions, such as the consecutive dehydrogenation of four methane molecules and the deoxygenation of two molecules of CO_2_, are consistent with previous experiments on the reaction of tantalum cations with methane and carbon dioxide.[^11b^, ^19^]

Analogously to the atomic cation, both molecules, CH_4_ and CO_2_, react in parallel with Ta_4_^+^. The reaction scheme, shown in Figure 5(D), is even more complex than the chemistry of Ta^+^, since the tantalum tetramer offers more oxidation steps than its monomeric analogue.^[19]^ Furthermore, it also exhibits different reactions like those involving intact adsorption. Despite the complex reaction scheme, the chemistry of [Ta_4_CH_2_]^+^+CO_2_ is clearly identified again. Consequently, for both species, the cationic atom and the tetramer, the coupling reaction also occurs in mixtures of the two reactants.

## Conclusion

In summary, the tantalum‐mediated coupling reactions exhibit a more complex pattern than described previously.[^6a^, ^14^] In addition to the observations made by Wesendrup and Schwarz in single collision experiments,^[6a]^ the present experiment under increased pressure reveals a second reaction channel for a C−C coupling reaction for the atomic cation. In the pathway reported in the literature,^[6a]^ TaCH_2_^+^, the product from methane dehydrogenation further reacts with carbon dioxide to form a Ta(O)CH_2_^+^ intermediate. Another CO_2_ molecule then enables the C−C coupling and the release of neutral C_2_H_2_O. The present experiments at elevated pressures confirm this pathway but also demonstrate a direct coupling of isolated TaCH_2_^+^ with CO_2_ with a similar ion yield as the two‐step process. We attribute this to different thermalization conditions in comparison to single collision studies. Surprisingly, the C−C coupling is completely suppressed when the reaction is mediated by tetrameric Ta_4_^+^ cation. In this case, only [Ta_4_CH_2_]^+^ reacts with CO_2_ to release a neutral “CH_2_O” unit, which is either formaldehyde, CO+H_2_, or a mixture thereof. Most likely the difference in the reactivity of the tetramer and the atomic cation is caused by the different structures of [Ta_4_CH_2_]^+^ (carbyne or carbide) compared to TaCH_2_^+^ (carbene). The corresponding DFT analysis demonstrates that the [Ta_4_CH_2_]^+^ ion taking part in the coupling reaction most likely features the carbyne hydride structure, i. e., HTa_4_CH^+^. While both, the carbyne hydride and the carbide dihydride, would show an exergonic conversion to the carbide oxide structure under exposure of CO_2_, the combination of a high desorption barrier of formaldehyde and a mandatory hydrogen diffusion step in case of H_2_ formation indicate that H_2_Ta_4_C^+^ is less likely to support the investigated reaction step than HTa_4_CH^+^.

Besides of the structural differences of the methane dehydrogenation products, it must be noted that DFT results from our previous study suggest that Ta^+^ is the only size of bare Ta clusters, which enables C−C coupling in consecutive reactions with further CH_4_ molecules.^[11b]^ Consequently, it may be speculated whether Ta_2_^+^ and Ta_3_^+^, whose intensities were too low to be studied, may also exhibit a reaction scheme related to Ta_4_^+^ in the reaction of CH_4_ and CO_2_. A reason for this peculiar reactivity of the atomic cation may be that the lack of additional Ta atoms does not enable the carbon to bind to further metal atoms as it is the case in larger system such as Ta_4_^+^. This study clearly demonstrates that the reaction of methane and CO_2_ undergoes completely different pathway when going from single atoms to clusters. While single atoms support products from C−C coupling reactions, species with more Ta atoms instead may rather yield molecules from C−O bond formations.

## Experimental Section

Tantalum cations are created in a laser ablation cluster source. For investigations of methane dehydrogenation products, the clusters are additionally modified chemically by the exposure to CH_4_ in a second reaction volume in the cluster source. The cations formed are then guided by electrostatic lenses and separated from neutrals by a quadrupole bender. A quadrupole mass filter enables the selection of particular cluster sizes prior to their eventual storage in a gas‐filled ring electrode ion trap. In the trap the ions are thermalized by collisions with the buffer gas (here: He) and exposed to the reactants (either CO_2_ or mixtures of CO_2_ and CH_4_). The tuning of the clusters’ storage time, which directly corresponds to the tuning of the reaction time, enables the elucidation of reaction kinetics. The qualitative and quantitative analysis of the charged species from the trap is performed with a reflectron time‐of‐flight mass spectrometer. Rate coefficients are obtained by modelling pseudo first‐order reaction kinetics of the experimental data with a home‐written computer program. (Further details are given in the Supporting Information.)

## Conflict of interest

The authors declare no conflict of interest.

1

## Supporting information

As a service to our authors and readers, this journal provides supporting information supplied by the authors. Such materials are peer reviewed and may be re‐organized for online delivery, but are not copy‐edited or typeset. Technical support issues arising from supporting information (other than missing files) should be addressed to the authors.

Supporting Information

## Data Availability

The data that support the findings of this study are available from the corresponding author upon reasonable request.
