# Supplementary material for: Direct Coupling of Methane and Carbon Dioxide on Tantalum Cluster Cations
Source: Chemistry. 2022 Dec 27;29(9):e202203259. doi: 10.1002/chem.202203259 (PMC10107500; doi:10.1002/chem.202203259)
Supplement: Supplementary file 1 — Supporting Information [file CHEM-29-0-s001.pdf]

Chemistry–A European Journal

Supporting Information

Direct Coupling of Methane and Carbon Dioxide on Tantalum Cluster Cations

Jozef Lengyel, Nikita Levin, Milan Ončák, Konstantin Jakob, Martin Tschurl,* and Ueli Heiz

I. Experimental details

The experiments are performed on the cluster beam setup, equipped with a cryogenic ring electrode ion trap in order to investigate ion-molecule reactions under multi-collisional conditions. A detailed description of the instrumental setup is found elsewhere.[1] Briefly, bare tantalum cluster cations are generated in a Smalley-type cluster source by laser vaporization (532 nm, 100 Hz, ~20 mJ pulse^{-1}) of a solid tantalum target, and the produced plasma is subsequently cooled in a pulse of He (Westfalen, 6.0) carrier gas.[2] To generate $[Ta_{1,4},C,2H]^+$, an additional pulse of methane (Rießner-Gase, 5.5) is introduced into the expansion channel of the source, followed by clustering via jet expansion into vacuum.[3] The product distribution of this reaction is controlled by changing the formation conditions, particularly by optimization of the CH_4 pulse. The skimmed cluster ions are transferred via electrostatic lens systems and a quadrupole bender into a quadrupole mass filter, selecting ions of interest, i.e., $Ta_{1,4}^+$ or $[Ta_{1,4},C,2H]^+$. The cluster ions are then guided into the ring electrode ion trap, where they are stored and thermalized close to ambient temperature (i.e., 300 K) through collisions with the He buffer gas. The reactant gas is continuously introduced into the trap at a total pressure of 0.82 Pa. Here, CO_2 mixed into the helium (25 – 200 ppm) is used in experiments with $[Ta_{1,4},C,2H]^+$, whereas CO_2/CH_4/He mixtures are used in reactions with bare $Ta_{1,4}^+$ clusters. The exact composition of these mixtures is given in the figure captions of the respective reactions. The absolute pressure in the trap is measured with a capacitance gauge (MKS, Baratron). For every measurement point, ions from five cycles of cluster generation are stored to ensure a good signal-to-noise ratio even for longer storage times. Hence, the accumulation time of ions corresponds to 5 ms, after which the nominal reaction time was chosen to be set to zero seconds. After chosen times, the reactant and product ions are transferred to and analyzed in a reflectron time-of-flight mass spectrometer (resolution of $M/\Delta M \approx 2000$). The reactions are monitored by recording mass spectra as a function of storage time. The progression of the reaction is modeled by kinetic simulations in order to obtain the rate coefficients of the reactions. Different reaction models are applied to achieve the best match between the calculated and measured progression.

II. RATE COEFFICIENTS

Table S1: Bimolecular rate coefficients $k^{(2)}$ in units of 10^{-9} cm^3s^{-1} of the reactions between isolated $TaCH_2^+$ and CO_2. Three different measurement series are averaged for each reaction with error bars reflecting corresponding statistical uncertainties. Each measurement series consists of four independent experiments at different concentrations of CO_2 in the buffer gas, namely 25, 50, 100, and 200 ppm; the total pressure in the trap is 0.82 Pa.

Reaction: $TaCH_2^+ + CO_2$	$k^{(2)}$ / 10^{-9} cm^3s^{-1}
$TaCH_2^+ + CO_2 \rightarrow TaO^+ + C_2H_2O$	1.8 ± 1.1
$TaCH_2^+ + CO_2 \rightarrow Ta(O)CH_2^+ + CO$	9.0 ± 2.3
$TaO^+ + CO_2 \rightarrow TaO_2^+ + CO$	7.1 ± 4.6
$Ta(O)CH_2^+ + CO_2 \rightarrow TaO_2^+ + C_2H_2O$	4.2 ± 2.4
$Ta(O)CH_2^+ + CO_2 \rightarrow Ta(O)CH_2(CO_2)^+$	9.2 ± 4.9
$Ta(O)CH_2CO_2^+ + CO_2 \rightarrow Ta(O)CH_2\,(CO_2)_2^+$	8.6 ± 2.8

Table S2: Bimolecular rate coefficients $k^{(2)}$ in units of 10^{-9} cm^3s^{-1} of the reactions between isolated $[Ta_4CH_2]^+$ and CO_2. Two measurement series are averaged for each reaction with error bars reflecting corresponding statistical uncertainties. Each measurement series consists of four independent experiments at different concentrations of CO_2 in the buffer gas, namely 25, 50, 100, and 200 ppm; the total pressure in the trap is 0.82 Pa.

Reaction: $Ta_4CH_2^+ + CO_2$	$k^{(2)}$ / 10^{-9} cm^3s^{-1}
$Ta_4CH_2^+ + CO_2 \rightarrow Ta_4(C)O^+ + CH_2O$	13.1 ± 2.6
$Ta_4(C)O^+ + CO_2 \rightarrow Ta_4(C)O_2^+ + CO$	13.4 ± 2.7
$Ta_4(C)O_2^+ + CO_2 \rightarrow Ta_4(C)O_3^+ + CO$	11.9 ± 2.1
$Ta_4(C)O_3^+ + CO_2 \rightarrow Ta_4(C)O_4^+ + CO$	9.3 ± 2.3
$Ta_4(C)O_4^+ + CO_2 \rightarrow Ta_4(C)O_5^+ + CO$	10.3 ± 3.0
$Ta_4(C)O_5^+ + CO_2 \rightarrow Ta_4(C)O_6^+ + CO$	3.6 ± 1.4
$Ta_4(C)O_6^+ + CO_2 \rightarrow Ta_4(C)O_7^+ + CO$	4.7 ± 2.2
$Ta_4(C)O^+ + CO_2 \rightarrow Ta_4(C)O(CO_2)^+$	2.9 ± 2.3
$Ta_4(C)O_2^+ + CO_2 \rightarrow Ta_4(C)O_2(CO_2)^+$	1.6 ± 0.8
$Ta_4(C)O_3^+ + CO_2 \rightarrow Ta_4(C)O_3(CO_2)^+$	0.7 ± 0.5
$Ta_4(C)O_4^+ + CO_2 \rightarrow Ta_4(C)O_4(CO_2)^+$	<0.1
$Ta_5(C)O_4^+ + CO_2 \rightarrow Ta_4(C)O_5(CO_2)^+$	2.9 ± 1.6

Table S3: Bimolecular rate coefficients $k^{(2)}$ in units of 10^{-9} cm^3 s^{-1} of the reactions between isolated Ta^+ with CH_4 and CO_2. For each reaction an average value of at least three individual measurements is presented with error bars reflecting corresponding statistical uncertainties. Different ratios of CH_4 to CO_2 in the buffer gas (1, 4.3, 5, 6.7, and 8.6) are used. Similar results for $k^{(2)}$ for the same reaction in the different measurements on the one hand support the pseudo-first order assumption and, on the other hand, enable the averaging of the obtained values. The exact concentrations of CO_2 vary between 35 and 100 ppm, fractions of methane between 50 and 300 ppm. The total pressure in the trap is 0.82 Pa.

Reaction: Ta^+ + CH_4 + CO_2	$k^{(2)}$ / 10^{-9} cm^3s^{-1}
$Ta^+ + CH_4 \rightarrow TaCH_2^+ + H_2$	1.5 ± 0.8
$TaCH_2^+ + CH_4 \rightarrow Ta(CH_2)_2^+ + H_2$	1.0 ± 0.3
$Ta(CH_2)_2^+ + CH_4 \rightarrow Ta(CH_2)_3^+ + H_2$	1.9 ± 0.6
$Ta(CH_2)_3^+ + CH_4 \rightarrow Ta(CH_2)_4^+ + H_2$	0.14 ± 0.03
$Ta^+ + CO_2 \rightarrow TaO^+ + CO$	5.8 ± 2.3
$TaO^+ + CO_2 \rightarrow TaO_2^+ + CO$	5.0 ± 1.3
$TaO^+ + CH_4 \rightarrow Ta(O)CH_2^+ + H_2$	1.3 ± 1.1
$Ta(O)CH_2^+ + CO_2 \rightarrow TaO_2^+ + C_2H_2O$	20.3 ± 11.4
$TaO_2^+ + CH_4 \rightarrow TaO_2(CH_4)^+$	2.4 ± 1.4
$TaCH_2^+ + CO_2 \rightarrow TaO^+ + C_2H_2O$	19.8 ± 7.0
$Ta(O)CH_2^+ + CO_2 \rightarrow Ta(O)CH_2(CO_2)^+$	6.3 ± 1.5
$TaO_2(CH_4)^+ + CO_2 \rightarrow TaO_2(CH_4)(CO_2)^+$	12.4 ± 6.7
$Ta(O)CH_2(CO_2)^+ + CO_2 \rightarrow Ta(O)CH_2(CO_2)_2^+$	15.4 ± 9.7
$Ta(CH_2)_2^+ + CO_2 \rightarrow Ta(O)(CH_2)_2^+ + CO$	3.5 ± 3.1

Table S4: Bimolecular rate coefficients $k^{(2)}$ in units of 10^{-9} cm^3 s^{-1} of the reactions between isolated Ta_4^+ with CH_4 and CO_2. For most of the reactions an average value of two individual measurements is presented with error bars reflecting corresponding statistical uncertainties. The ratios of CH_4 to CO_2 in the buffer gas are constant with a value of $[CH_4]:[CO_2]$ equal to 100. The exact values are either 25 or 50 ppm for CO_2, and 2500 or 5000 ppm for CH_4. As similar results were obtained for $k^{(2)}$ for the same reaction in the different measurements, evidence for the pseudo-first order assumption is supplied and averaging of the obtained values becomes possible. The total pressure in the trap is 0.82 Pa.

Reaction: Ta_4^+ + CH_4 + CO_2	$k^{(2)}$ / 10^{-9} cm^{3}s^{-1}
$Ta_4^+ + CH_4 \rightarrow Ta_4CH_2^+ + H_2$	0.02 ± 0.01
$Ta_4CH_2^+ + CH_4 \rightarrow Ta_4(CH_2)_2^+ + H_2$	0.014 ± 0.001
$Ta_4CH_2^+ + CO_2 \rightarrow Ta_4(C)O^+ + H_2CO$	0.67 ± 0.63
$Ta_4(C)O^+ + CO_2 \rightarrow Ta_4(C)O_2^+ + CO$	8.6 ± 3.0
$Ta_4(C)O_2^+ + CO_2 \rightarrow Ta_4(C)O_3^+ + CO$	3.0 ± 2.0
$Ta_4(C)O_3^+ + CO_2 \rightarrow Ta_4(C)O_4^+ + CO$	4.8 ± 3.2
$Ta_4(C)O_4^+ + CO_2 \rightarrow Ta_4(C)O_5^+ + CO$	3.0 ± 0.6
$Ta_4^+ + CO_2 \rightarrow Ta_4O^+ + CO$	2.6 ± 0.9
$Ta_4O^+ + CO_2 \rightarrow Ta_4O_2^+ + CO$	1.9 ± 0.7
$Ta_4O^+ + CH_4 \rightarrow Ta_4(O)CH_2^+ + H_2$	0.04 ± 0.01
$Ta_4(O)CH_2^+ + CH_4 \rightarrow Ta_4(O)(CH_2)_2^+ + H_2$	0.04 ± 0.02
$Ta_4CH_2^+ + CO_2 \rightarrow Ta_4CH_2(CO_2)^+$	11.0 ± 5.0
$Ta_4CH_2(CO_2)^+ + CO_2 \rightarrow Ta_4CH_2(CO_2)_2^+$	5.0 ± 1.8
$Ta_4O^+ + CO_2 \rightarrow Ta_4O(CO_2)^+$	0.34 ± 0.08
$Ta_4O_2^+ + CO_2 \rightarrow Ta_4O_2(CO_2)^+$	9.1 ± 9.1
$Ta_4(C)O^+ + CO_2 \rightarrow Ta_4(C)O(CO_2)^+$	3.9 ± 2.0
$Ta_4(C)O_2^+ + CO_2 \rightarrow Ta_4(C)O_2(CO_2)^+$	3.4 ± 1.7
$Ta_4(O)CH_2^+ + CO_2 \rightarrow Ta_4(O)CH_2(CO_2)^+$	2.4 ± 1.7

III. Kinetic modeling of the coupling on bare clusters

This section provides examples of results from the kinetic fitting procedure, from which the rate coefficients (see Tables S3 and S4) of different reactions of bare Ta^+ and Ta_4^+ and their intermediates with carbon dioxide and methane are obtained.

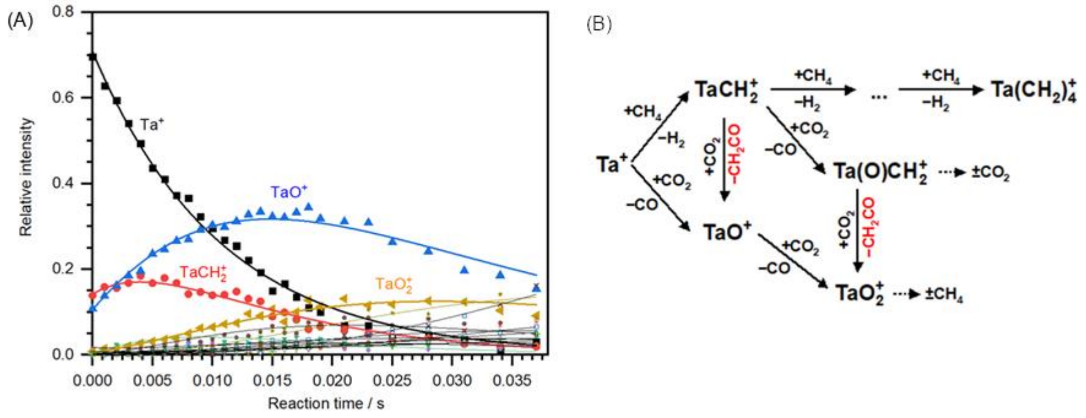

Figure S1: Kinetic analysis and the underlying mechanism of the mass-selected Ta^+ ions with a mixture of CO_2 and CH_4 measured at 300 K. The experimental data points correspond to the normalized intensities of the reactants, intermediates, and products. The total pressure in the trap amounts to 0.82 Pa, the proportion of CO_2 in the mixture is 20 ppm and of CH_4 100 ppm.

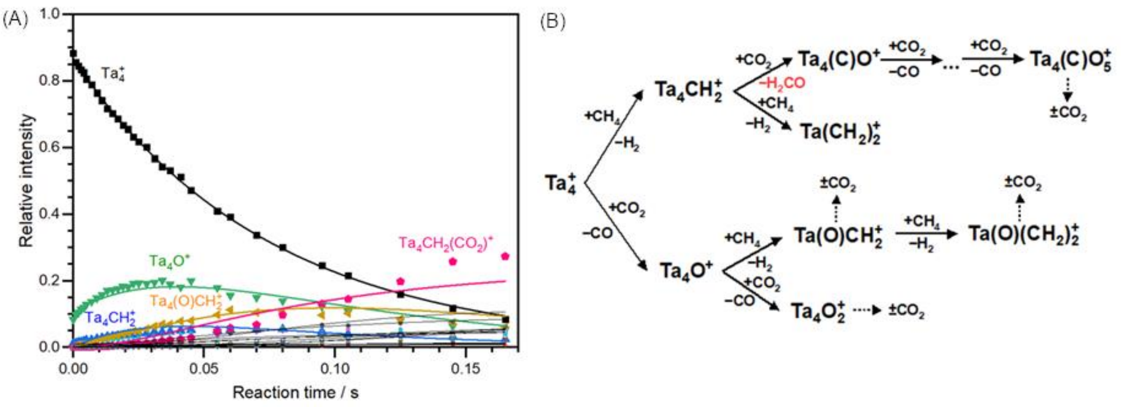

Figure S2: Kinetic analysis and the underlying mechanism of the mass-selected Ta_4^+ ions with a mixture of CO_2 and CH_4 measured at 300 K. The experimental data points correspond to the normalized intensities of the reactants, intermediates, and products. The total pressure in the trap amounts to 0.82 Pa, the proportion of CO_2 in the mixture is 25 ppm and of CH_4 2500 ppm.

IV. ABUNDANCE OF TA_N^+ CLUSTERS GENERATED IN THE CLUSTER SOURCE

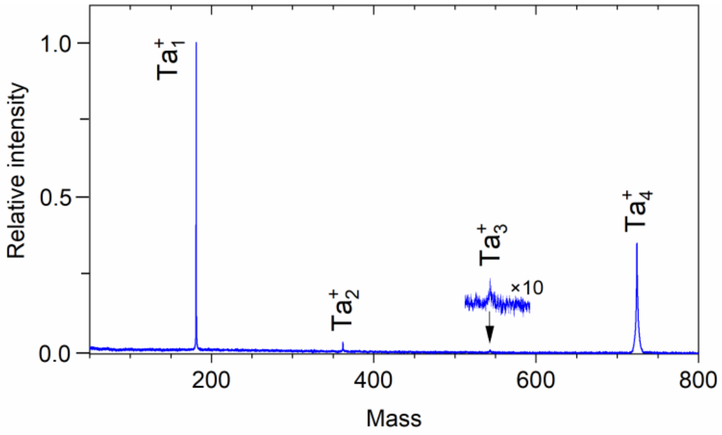

Figure S3: Typical spectra of Ta_{1-4}^+ clusters generated by a laser vaporization source. The relative abundance of the different sizes is in excellent agreement with the work of Sakurai et al.,[4] for which a spectrum is shown up to Ta_{40}^+.

V. Calculation Details, Hydrogen Desorption on $H_2Ta_4C^+$ Clusters and Full Reaction Pathways from Figure 3

All isomers were first optimized at the HSE06/def2TZVP level of theory, with subsequent frequency calculation to assess the nature of the stationary point (local minimum or transition state). In the optimized structures, a single-point CCSD/def2TZVP calculation was performed to obtain more reliable energies. Due to the single-point recalculations at a different level of theory, the energy of close-lying local minima and transition states might be inverted, i.e., transition states might lie slightly below the respective minima. Zero-point and thermochemical corrections were applied as calculated at the HSE06/def2TZVP level. All energies reported in the manuscript are zero-point corrected energies at 0 K, unless stated otherwise. Enthalpies and Gibbs energies were calculated at 298.15 K and 1 bar. Wave function stabilization was performed prior to every calculation.

In order to supply additional indications whether the desorbing product "CH_2O" is either formaldehyde or syngas, a hypothetical hydrogen production route is evaluated. The appearance of the Ta_4C^+ may be either indicative for the release of H_2 (and thus a formation pathway yielding syngas) or the result of insufficient mass selection. For hydrogen to desorb, the hydrogen atoms need to get close enough together to be able to form an H−H bond. The hydrogen mobility can be estimated by calculating the activation barrier for a hydrogen atom under a change of the adsorption site. This diffusion process is shown exemplarily in Figure S4 in the case of carbide dihydride ($H_2Ta_4C^+$). The proposed dissociation pathways shows a barrier of 1.39 eV for H_2 formation that however lies below the dissociation energy of 1.49 eV. As an alternative, the energy of CO_2 adsorption might be used to initiate the reaction, but the kinetic fits (see Figure 2) reveal that the resulting product species $[Ta_4C(CO_2)]^+$ rather originates from an uptake of CO_2 by Ta_4C^+.

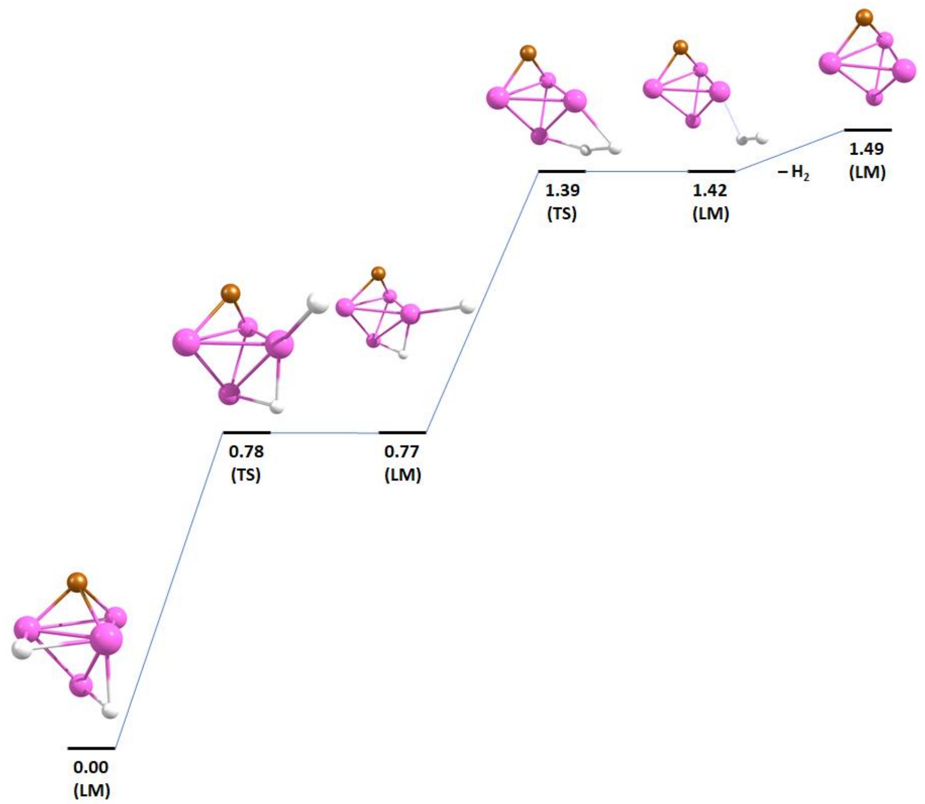

Figure S4: A proposed pathway for hydrogen molecule formation and desorption from $H_2Ta_4C^+$. Calculated at the CCSD/def2TZVP//HSE06/def2TZVP level, energies are given in eV. Local minima (LM) might lie above transition states (TS) due to single-point CCSD recalculation.

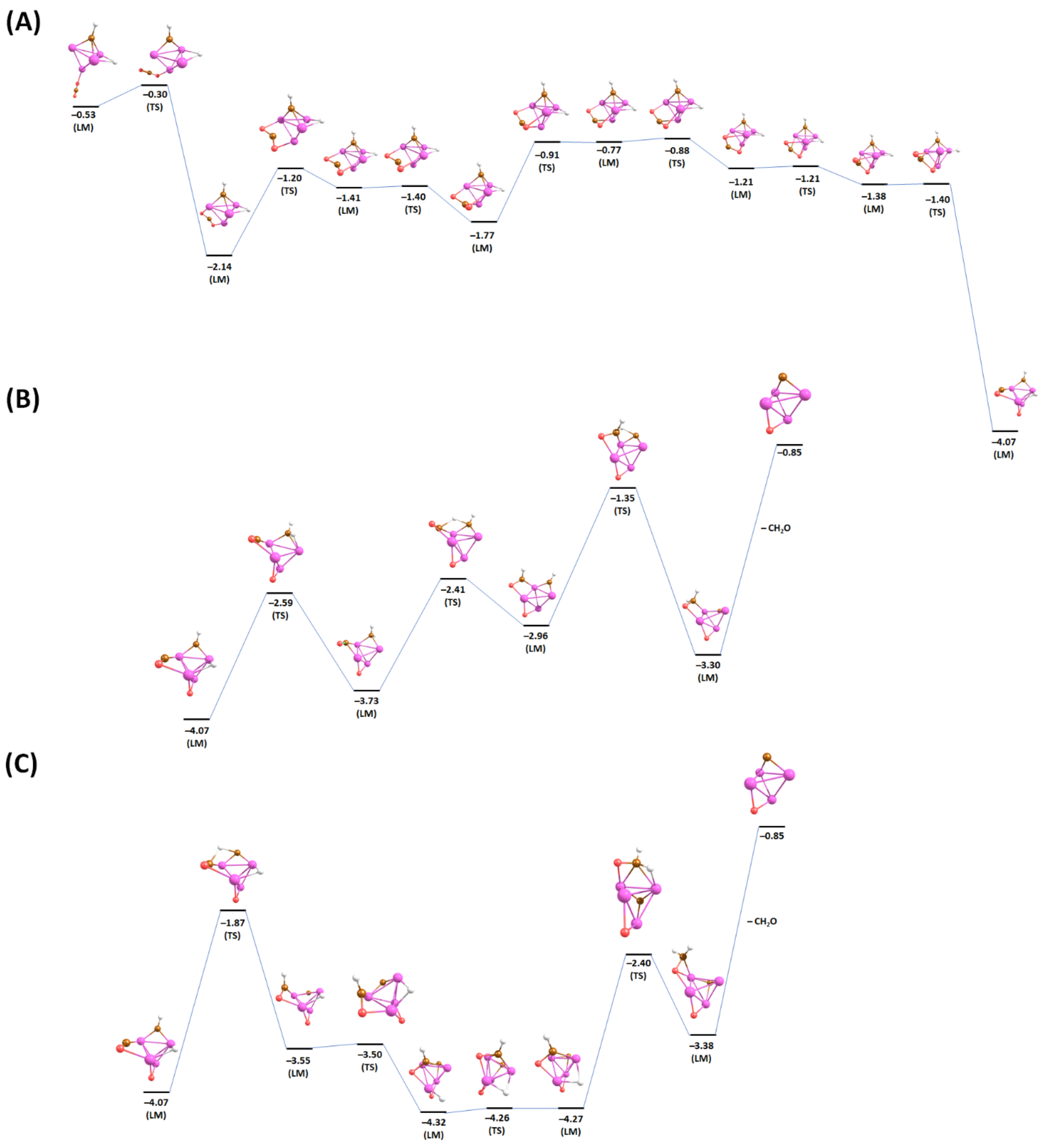

Figure S5: The full pathway for reaction depicted in Figure 3C, from CO_2 adsorption to C–O bond dissociation (A), followed by two different pathways for CH_2O formation and desorption (B,C). Calculated at the CCSD/def2TZVP//HSE06/def2TZVP level, energies are given in eV. Local minima (LM) might lie above transition states (TS) due to single-point CCSD recalculation.

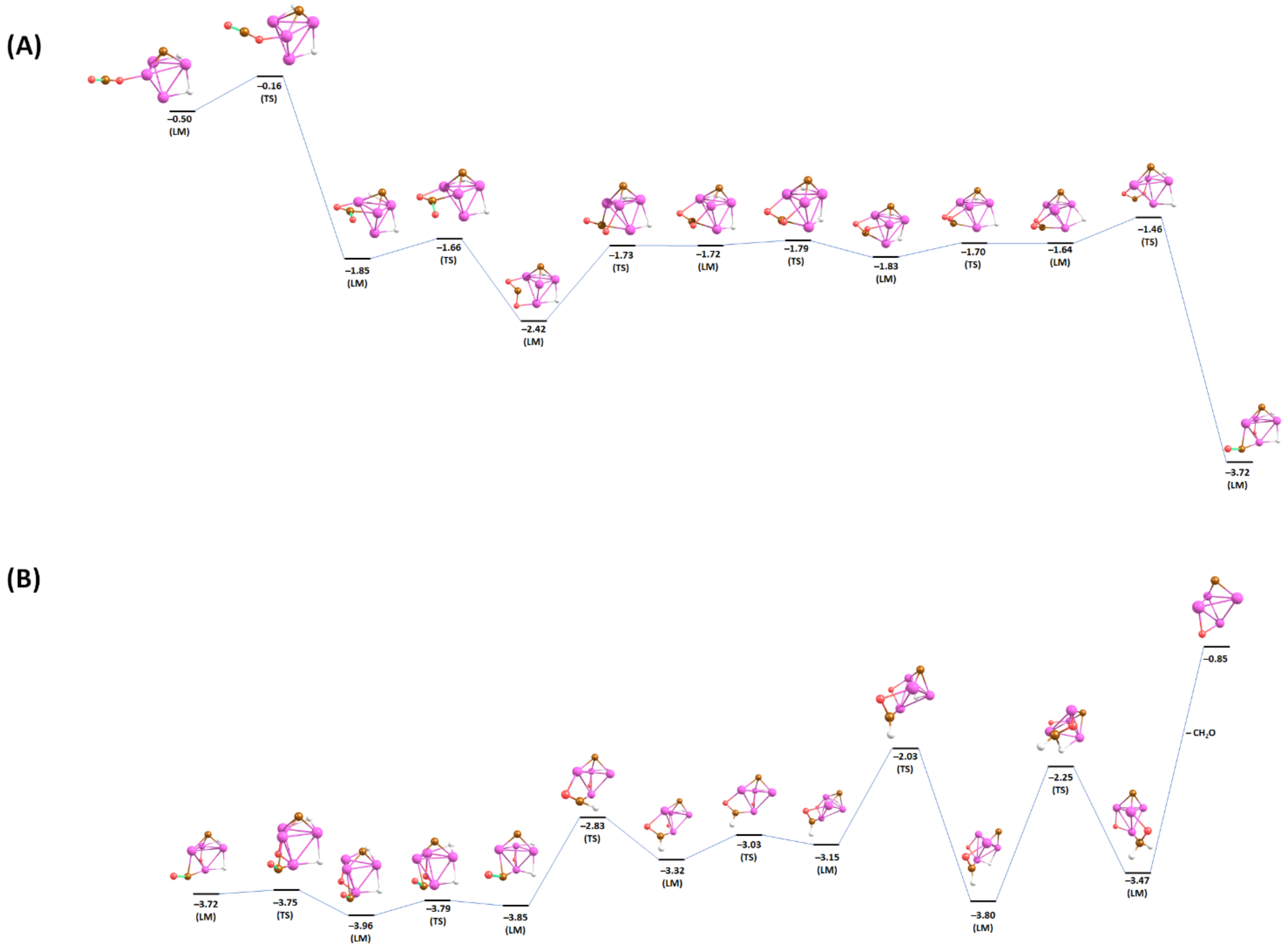

Figure S6: The full pathway for reaction depicted in Figure 3D, from CO_2 adsorption to C–O bond dissociation (A), followed by a pathway for CH_2O formation and desorption (B). Calculated at the CCSD/def2TZVP//HSE06/def2TZVP level, energies are given in eV. Local minima (LM) might lie above transition states (TS) due to CCSD single-point recalculation.

Table S5: Zero-point corrected energies *E* at 0 K, and enthalpies *H* and Gibbs energies *G* at 298.15 K and 1 atmosphere (all in eV) for local minima and transition states shown in Figure 3.

point	singlet			triplet		
	E	*H*	*G*	*E*	*H*	*G*
LM	0.05	0.04	0.07	0.00	0.00	0.00
LM	-0.83	-0.83	-0.47	-0.95	-0.95	-0.62
TS	-0.29	-0.32	0.13	-0.03	-0.05	0.37
LM	-3.24	-3.26	-2.81	-2.27	-2.27	-1.91
LM	-1.51	-1.50	-1.50	-1.22	-1.20	-1.25
LM	-2.93	-2.92	-2.55	-2.47	-2.43	-2.18
TS	-2.41	-2.44	-1.95	-1.00	-1.00	-0.60
LM	-2.92	-2.93	-2.51	-1.25	-1.26	-0.83
TS	-2.93	-2.95	-2.48	-0.97	-0.99	-0.56
LM	-3.78	-3.78	-3.37	-1.93	-1.92	-1.57
LM	-0.90	-0.88	-0.94	0.59	0.64	0.48

LM	0.05	0.04	0.07	0.00	0.00	0.00
LM	-0.83	-0.83	-0.47	-0.95	-0.95	-0.62
TS	-0.20	-0.23	0.24	-0.37	-0.41	0.03
LM	-2.21	-2.23	-1.83	-2.44	-2.46	-2.08
LM	-0.32	-0.31	-0.29	-0.49	-0.49	-0.49
LM	-1.34	-1.34	-1.01	-1.58	-1.58	-1.28
TS	-0.69	-0.71	-0.31	0.71	0.70	1.06
LM	-2.62	-2.62	-2.26	-0.39	-0.38	-0.08
LM	-0.90	-0.88	-0.94	0.59	0.64	0.48

Table S6: Zero-point corrected energies *E* at 0 K, and enthalpies *H* and Gibbs energies *G* at 298.15 K and 1 atmosphere (all in eV) for local minima and transition states shown in Figure S4.

point	*E*	*H*	*G*
LM	0.00	0.00	0.00
TS	0.78	0.78	0.77
LM	0.77	0.79	0.75
TS	1.39	1.40	1.38
LM	1.42	1.44	1.40
LM	1.49	1.57	1.17

Table S7: Zero-point corrected energies *E* at 0 K, and enthalpies *H* and Gibbs energies *G* at 298.15 K and 1 atmosphere (all in eV) for local minima and transition states shown in Figure S5.

point	*E*	*H*	*G*
LM	-0.53	-0.51	-0.23
TS	-0.30	-0.30	0.12
LM	-2.14	-2.16	-1.66
TS	-1.20	-1.23	-0.70
LM	-1.41	-1.42	-0.93
TS	-1.40	-1.43	-0.90
LM	-1.77	-1.80	-1.28
TS	-0.91	-0.93	-0.42
LM	-0.77	-0.79	-0.30
TS	-0.88	-0.92	-0.36
LM	-1.21	-1.23	-0.70
TS	-1.21	-1.25	-0.69
LM	-1.38	-1.41	-0.87
TS	-1.40	-1.43	-0.89
LM	-4.07	-4.09	-3.58
TS	-2.59	-2.61	-2.11
LM	-3.73	-3.74	-3.24
TS	-2.41	-2.43	-1.93
LM	-2.96	-2.97	-2.48
TS	-1.35	-1.37	-0.87
LM	-3.30	-3.32	-2.83
TS	-1.87	-1.89	-1.40

LM	-3.55	-3.57	-3.06
TS	-3.50	-3.54	-2.99
LM	-4.32	-4.35	-3.82
TS	-4.26	-4.31	-3.74
LM	-4.27	-4.30	-3.76
TS	-2.40	-2.44	-1.89
LM	-3.38	-3.40	-2.90
LM	-0.85	-0.84	-0.87

Table S8: Zero-point corrected energies *E* at 0 K, and enthalpies *H* and Gibbs energies *G* at 298.15 K and 1 atmosphere (all in eV) for local minima and transition states shown in Figure S6.

point	*E*	*H*	*G*
LM	-0.50	-0.47	-0.18
TS	-0.16	-0.16	0.24
LM	-1.85	-1.87	-1.38
TS	-1.66	-1.70	-1.15
LM	-2.42	-2.45	-1.91
TS	-1.73	-1.77	-1.22
LM	-1.72	-1.74	-1.23
TS	-1.79	-1.83	-1.29
LM	-1.83	-1.86	-1.33
TS	-1.70	-1.75	-1.20
LM	-1.64	-1.67	-1.15
TS	-1.46	-1.49	-0.98
LM	-3.72	-3.73	-3.25
TS	-3.75	-3.78	-3.26
LM	-3.96	-3.98	-3.47
TS	-3.79	-3.81	-3.29
LM	-3.85	-3.87	-3.36
TS	-2.83	-2.87	-2.33
LM	-3.32	-3.34	-2.83
TS	-3.03	-3.07	-2.53
LM	-3.15	-3.17	-2.66
TS	-2.03	-2.05	-1.55
LM	-3.80	-3.82	-3.31
TS	-2.25	-2.27	-1.76
LM	-3.47	-3.49	-2.99
LM	-0.85	-0.84	-0.87

VIII. Cartesian coordinates (in Å) of structures optimized at the HSE06/def2TZVP level along with zero-point corrected energies (in a.u.)

```
CH2O
E = -114.402443
c -0.000000 0.000000 -0.524140
h -0.000000 -0.939656 -1.112043
h 0.000000 0.939656 -1.112043
o 0.000000 -0.000000 0.671116

CO2
E = -188.472076
C 0.000000 0.000000 0.000000
O 0.000000 0.000000 1.156563
O 0.000000 0.000000 -1.156563

Ta4CH2+
E = -266.927164
Ta -0.136835 1.432982 0.525638
Ta 0.205584 -1.030685 1.066426
Ta -1.579774 -0.264530 -0.659345
Ta 1.479268 0.013504 -0.815012
C 0.024991 -1.314738 -1.254663
H 0.058793 -2.276623 -1.781032
H 2.109566 -0.877758 0.716427

CH2CO
E = -152.470486
H 0.000000 0.939600 -1.735189
H -0.000000 -0.939600 -1.735189
C 0.000000 0.000000 -1.203618
C 0.000000 -0.000000 0.103040
O 0.000000 -0.000000 1.259231

CO
E = -113.236403
C 0.000000 0.000000 -0.641763
O 0.000000 0.000000 0.481322

Pathway from Figure 3a

LM1, S=0
E = -95.828624
Ta 0.011052 -0.190527 -0.000000
C 0.011052 1.631109 0.000000
H -1.127788 1.433624 0.000000
H 0.254661 2.688223 0.000000

LM2, S=0
E = -284.334153
Ta 0.660778 -0.179699 -0.005577
C 1.019195 1.610372 -0.069459
O -1.507172 -0.465383 0.000664
H 1.397623 1.345966 1.000242
H 1.111092 2.666836 -0.291870
C -2.597332 -0.011281 0.004821
O -3.652416 0.404217 0.010156

TS1, S=0
E = -284.322013
c -1.926548 -0.070752 0.032567
ta 0.540564 -0.143290 -0.079562
o -1.242746 -0.883557 0.654853
o -2.838390 0.508976 -0.338914
c 0.514405 1.648934 0.282242
h 0.265363 2.693874 0.426735
h 1.395436 1.293840 0.964904

LM3, S=0
E = -284.419892
Ta 0.386002 -0.074489 -0.199479
C 0.121315 1.707853 0.520304
O 0.572892 -0.911179 1.248964
H 0.092806 1.995572 1.568229
H -0.051169 2.544668 -0.168668
C -1.759754 -0.140801 -0.074333
O -2.871532 -0.151932 0.061856

LM4, S=0
E = -171.111096
Ta -0.000000 0.217948 -0.000000
O -1.398979 -0.699568 0.000000
C 1.287267 -1.192055 -0.000000
H 2.345884 -0.895965 -0.000000
H 1.122348 -2.265342 -0.000000

LM5, S=0
E = -359.633269
Ta 0.605802 -0.094488 -0.176040
C 0.877458 1.658567 0.534544
H 0.800146 2.483475 -0.187570
H 1.062484 1.975039 1.555975
O 0.709474 -0.996832 1.240712
O -1.534102 -0.086137 -0.547276
C -2.603109 0.024892 -0.057192
O -3.641909 0.125260 0.383868

TS2, S=0
E = -359.621655
c -1.807665 -0.217673 0.075670
c -0.743983 1.447688 -0.141908
ta 0.559293 -0.022238 -0.163800
o -1.059462 -1.224339 -0.160232
o 1.063463 -0.016259 1.436477
o -2.915159 0.042680 0.312797
h -1.221908 1.768055 -1.074190
h -1.007339 2.058552 0.716712
```

LM6, S=0
E = -359.635874
c -1.964774 0.045193 0.073131
c -1.153502 1.262277 -0.151910
ta 0.598124 -0.034408 -0.171400
o -0.899120 -1.068425 0.028459
o 1.176829 0.188423 1.393990
o -3.068424 -0.251971 0.259061
h -1.382229 1.728970 -1.117134
h -1.245443 1.993770 0.649943

TS3, S=0
E = -359.636248
c -1.970837 0.053873 0.058703
o -0.877558 -1.082300 -0.068705
c -1.160134 1.263119 -0.167515
o -3.053022 -0.270905 0.293836
ta 0.602954 -0.026982 -0.165360
o 1.108138 0.147532 1.432921
h -1.278667 2.014595 0.611486
h -1.371629 1.698528 -1.151761

LM7, S=0
E = -359.663467
Ta -0.622128 0.106087 -0.000046
O -0.254961 -0.880935 1.347196
O -0.254863 -0.881895 -1.346558
C 1.388499 1.201906 -0.000373
H 1.514422 1.796736 -0.911897
H 1.514405 1.797311 0.910777
C 2.309714 0.169729 -0.000041
O 3.034475 -0.683193 0.000233

LM8, S=0
E = -207.090749
Ta 0.000000 0.188009 0.000000
O 1.326130 -0.857789 -0.000000
O -1.326130 -0.857789 0.000000

LM1, S=1
E = -95.833367
Ta 0.010509 -0.192058 0.000000
C 0.010509 1.641807 -0.000000
H -1.125397 1.481245 0.000000
H 0.295181 2.688113 -0.000000

LM2, S=1
E = -284.341261
Ta 0.670125 -0.179090 -0.004898
C 0.939906 1.638748 -0.066885
O -1.502888 -0.467593 -0.004210
H 1.308248 1.433579 1.006568
H 0.985958 2.687051 -0.335537
C -2.595543 -0.025749 0.003868
O -3.657053 0.376957 0.012291

TS1, S=1
E = -284.313075
c 1.584257 0.179920 -0.118217
ta -0.444479 -0.195987 -0.089498
o 1.055822 -0.805738 0.821686
o 2.665439 0.551999 -0.283144
c -0.841786 1.759071 0.342098
h -0.984846 2.189220 1.334472
h -0.793079 2.513813 -0.452725

LM3, S=1
E = -284.381163
Ta 0.255745 -0.067170 -0.196804
C 1.609852 1.321700 0.580623
O 0.423060 -1.413728 0.797006
H 2.402653 1.043871 1.274736
H 1.569451 2.397788 0.395665
C -1.941098 0.242602 0.032577
O -3.004812 0.423217 0.330131

LM4, S=1
E = -171.098923
Ta 0.122096 -0.195036 -0.000000
O 0.868756 1.299433 0.000000
C -1.840780 0.437283 0.000000
H -2.679302 -0.265231 -0.000000
H -2.139086 1.483694 -0.000001

LM5, S=1
E = -359.612158
Ta 0.492423 0.004660 -0.039314
C 2.264991 -1.076354 0.160535
H 2.334895 -2.166663 0.156983
H 3.238904 -0.605154 0.288891
O 0.995067 1.601075 0.115981
O -1.714491 -0.316997 -0.056548
C -2.883628 -0.171392 0.026411
O -4.006682 -0.044312 0.103363

TS2, S=1
E = -359.567388
c -2.070090 -0.320281 0.122789
c -0.929337 1.505876 0.013732
ta 0.592842 -0.030043 -0.178178
o -1.157604 -1.145248 0.073337
o 1.482655 0.022057 1.246061
o -3.171922 0.003037 0.185459
h -1.311297 2.094018 -0.825172
h -1.194641 1.946783 0.974211

LM6, S=1
E = -359.584073
c -1.902593 -0.122826 0.106707
c -1.274567 1.180019 -0.378893
ta 0.628175 -0.074896 -0.160512
o -1.088027 -1.089094 0.299201
o 1.207691 0.583969 1.269365
o -3.102304 -0.039143 0.230335
h -1.589899 1.481346 -1.378317
h -1.342802 1.997008 0.337633

TS3, S=1
E = -359.567955
o -0.997425 -0.942026 0.068664
c -2.073974 -0.077235 0.002124
ta 0.722281 -0.135388 -0.061868
o 0.773765 1.290991 0.826923
c -1.674757 1.139018 -0.721032
o -3.132387 -0.308153 0.468978
h -1.486877 1.074995 -1.790867
h -1.898899 2.111171 -0.295813

LM7, S=1
E = -359.599229
o 0.956404 0.556945 0.131117
c 2.246578 -0.147438 0.069534
ta -0.816785 0.163075 0.029567
o -0.842463 -1.442171 -0.452220
c 3.291424 0.728070 -0.317473
o 2.261213 -1.298759 0.352093
h 3.110371 1.773239 -0.520790
h 4.285701 0.310358 -0.397873

LM8, S=1
E = -207.034823
Ta 0.000000 0.216238 -0.000000
O 1.258976 -1.000963 0.000000
O -1.258976 -0.972212 -0.000000

Pathway from Figure 3b

LM1, S=0
E = -95.828624
Ta 0.011052 -0.190527 -0.000000
C 0.011052 1.631109 0.000000
H -1.127788 1.433624 0.000000
H 0.254661 2.688223 0.000000

LM2, S=0
E = -284.334153
Ta 0.660778 -0.179699 -0.005577
C 1.019195 1.610372 -0.069459
O -1.507172 -0.465383 0.000664
H 1.397623 1.345966 1.000242
H 1.111092 2.666836 -0.291870
C -2.597332 -0.011281 0.004821
O -3.652416 0.404217 0.010156

TS1, S=0
E = -284.319166
c 1.793969 -0.252923 0.000000
c 0.399843 1.570836 0.000007
ta -0.625652 -0.048237 -0.000001
o 1.017799 -1.231498 0.000010
o 2.865677 0.165371 -0.000007
h 0.720977 2.071431 -0.919085
h 0.720977 2.071427 0.919101

LM3, S=0
E = -284.386614
Ta -0.656369 -0.074337 -0.055641
O -0.164848 1.470034 0.383027
C 1.347437 -1.119569 0.329739
H 1.416818 -1.367060 1.396989
H 1.463469 -2.007244 -0.302388
C 2.306374 -0.184219 0.005669
O 3.053821 0.607919 -0.263683

LM4, S=0
E = -131.841860
Ta 0.000000 0.000000 0.163799
O 0.000000 0.000000 -1.494665

LM5, S=0
E = -320.354635
Ta -0.000000 0.690409 0.000000
O 1.660997 0.515284 0.000000
O -0.748240 -1.322730 -0.000000
C -0.591964 -2.495378 -0.000000
O -0.468783 -3.621004 -0.000000

TS2, S=0
E = -320.339214
o 1.225497 -0.588026 0.864097
ta -0.519933 -0.138782 -0.095592
c 1.947530 0.004706 0.054606
o 2.887753 0.427907 -0.435649
o -0.829511 1.422979 0.402873

LM6, S=0
E = -320.393290
Ta -0.385149 -0.000000 -0.189756
O -0.436164 -1.338582 0.863991
O -0.436174 1.338580 0.863992
C 1.879685 0.000003 -0.092656
O 2.977061 0.000003 0.073032

LM7, S=0
E = -207.090749
Ta 0.000000 0.188009 0.000000
O 1.326130 -0.857789 -0.000000
O -1.326130 -0.857789 0.000000

LM1, S=1
E = -95.833367
Ta 0.010509 -0.192058 0.000000
C 0.010509 1.641807 -0.000000
H -1.125397 1.481245 0.000000
H 0.295181 2.688113 -0.000000

LM2, S=1
E = -284.341261
Ta 0.670125 -0.179090 -0.004898
C 0.939906 1.638748 -0.066885
O -1.502888 -0.467593 -0.004210
H 1.308248 1.433579 1.006568
H 0.985958 2.687051 -0.335537
C -2.595543 -0.025749 0.003868
O -3.657053 0.376957 0.012291

TS1, S=1
E = -284.327133
c -1.809982 -0.256228 0.000002
c -0.379519 1.586529 0.000003
ta 0.627957 -0.051546 -0.000000
o -1.042076 -1.235213 0.000002
o -2.873314 0.182957 -0.000003
h -0.690390 2.099531 0.915798
h -0.690369 2.099535 -0.915798

LM3, S=1
E = -284.397000
Ta -0.652678 -0.076453 -0.061967
O -0.190913 1.464470 0.427771
C 1.360992 -1.114443 0.367787
H 1.442420 -1.335318 1.438546
H 1.484403 -2.015240 -0.243848
C 2.302199 -0.176524 0.008842
O 3.033357 0.620205 -0.294129

LM4, S=1
E = -131.850713
Ta 0.000000 0.000000 0.163941
O 0.000000 0.000000 -1.495965

LM5, S=1
E = -320.365454
Ta 0.000000 0.694681 0.000000
O 1.660908 0.498852 0.000000
O -0.746546 -1.331465 0.000000
C -0.592012 -2.502618 0.000000
O -0.470352 -3.629387 0.000000

TS2, S=1
E = -320.277981
o 0.870300 -1.239581 0.716970
ta -0.473436 -0.088676 -0.114456
c 1.698599 0.237049 -0.189075
o 2.795506 0.454762 -0.171821
o -0.619656 1.416205 0.641066

LM6, S=1
E = -320.316071
Ta -0.289921 0.016520 -0.190210
O -1.276096 -1.268570 0.737599
O -0.549268 1.413081 0.723834
C 1.941274 -0.113277 -0.010950
O 3.014934 -0.210296 0.282448

LM7, S=1
E = -207.034823
Ta 0.000000 0.216238 -0.000000
O 1.258976 -1.000963 0.000000
O -1.258976 -0.972212 -0.000000

Pathway from Figure S4

LM1
E = -266.916303
h -2.215230 0.682693 0.655978
ta -1.406979 -0.180209 -0.836290
ta 1.532427 0.405616 -0.636260
ta -0.372114 1.130457 0.959394
ta 0.287984 -1.464830 0.576469
h -0.092131 -0.380814 2.057193
c -0.118149 1.275442 -1.222511

TS1
E = -266.879272
h -3.158784 0.001877 0.980269
ta -0.354044 -1.261451 -0.911335
ta 0.426499 1.439623 -0.663894
ta -1.348506 0.255572 0.883031
ta 1.411646 -0.472978 0.757589
h 0.081737 -0.217616 2.074429
c -1.136901 0.513294 -1.304700

LM2
E = -266.880492
h 3.144583 0.000047 1.483010
h 0.012627 -0.000006 2.057490
ta -1.428773 -0.000038 0.843870
ta 1.443511 0.000030 0.796375
ta -0.077363 1.412951 -0.789038
ta -0.077290 -1.412947 -0.789049
c 1.176089 0.000039 -1.346347

TS2
E = -266.868169
h 0.003565 0.395366 3.015898
h -0.000022 1.040235 2.172092
ta -0.003592 1.612478 0.069199
ta 0.002351 -0.636528 1.410850
ta 1.382021 -0.421490 -0.746705
ta -1.381112 -0.427398 -0.745018
c 0.003446 -1.785191 -0.722631

LM3
E = -266.866906
h 0.000898 0.983729 3.072191
h 0.001110 1.436538 2.363570
ta 0.000765 1.601761 -0.234248
ta -0.000044 -0.336645 1.486390
ta 1.374666 -0.571971 -0.652149
ta -1.375337 -0.570658 -0.651939
c -0.000951 -1.893622 -0.273953

LM4
E = -265.699306
ta 0.000239 1.621989 0.029257
ta -0.000125 -0.541980 1.419864
ta 1.379739 -0.465217 -0.706797
ta -1.379834 -0.464828 -0.706865
c -0.000239 -1.824575 -0.431421

H2
E = -1.158295

h 0.000000 0.000000 0.372863
h 0.000000 0.000000 -0.372863

Pathway from Figure S5

LM1
E = -455.414218
c -4.758583 -0.041122 0.148591
ta 0.779676 1.608513 -0.508503
o -3.608141 0.032285 -0.052554
o -5.881791 -0.112219 0.342893
ta -1.191954 0.059900 -0.118548
ta 0.867555 -0.172928 1.383657
ta 0.749758 -1.461690 -0.754128
c 2.061850 0.067550 -0.617771
h 3.156014 0.089785 -0.701222
h 0.976312 -2.075947 1.012640

TS1
E = -455.408047
c -3.538522 -0.619189 -0.074794
ta -0.793226 1.286524 -0.576030
o -3.063080 -1.583023 0.413353
o -4.149705 0.238814 -0.539010
ta -0.752316 -0.976893 0.574585
ta 1.138883 0.705370 1.032436
ta 1.342426 -0.917788 -0.887109
c 1.069531 1.033886 -1.290159
h 1.691503 1.749589 -1.842247
h 2.513699 -0.580628 0.533861

LM2
E = -455.467553
c 2.468448 0.576175 -0.182165
ta 1.140393 -1.137256 -0.397002
o 2.667852 1.758800 0.143962
o 3.126376 -0.363227 -0.698913
ta 0.546752 1.237934 0.575686
ta -1.063020 -0.747954 1.052755
ta -1.351048 0.582815 -1.040018
c -0.714918 -1.348329 -1.176567
h -1.257325 -2.105002 -1.755990
h -2.552362 0.278962 0.374224

TS2
E = -455.437253
o -1.393786 2.317388 -0.240274
ta -0.261775 0.639591 1.328491
ta 0.875704 1.108656 -0.845738
ta 1.266770 -1.115894 0.225478
ta -1.329676 -0.981275 -0.374132
c -1.777884 1.136118 -0.336189
o -2.759263 0.567292 -0.886315
c 0.145215 -0.617479 -1.690684
h 0.306316 -0.961558 -2.717527
h 2.489383 0.243603 -0.497747

LM3
E = -455.440261
o -0.991045 2.532156 -0.314484
ta -0.163266 0.255546 1.473920
c -1.545705 1.420323 -0.215074
o -2.673316 1.007091 -0.597509
ta -1.428452 -0.774518 -0.475831
ta 0.897406 1.251304 -0.587444
ta 1.185010 -1.217095 -0.102992
c 0.032743 -0.216080 -1.751515
h 0.099596 -0.336045 -2.838159
h 2.472139 0.184283 -0.525064

TS3
E = -455.440010
o -0.603547 2.626124 -0.519957
ta -0.284659 0.362752 1.422803
c -1.288878 1.610954 -0.236850
o -2.418437 1.267543 -0.698729
ta -1.465063 -0.676662 -0.539077
ta 1.080936 1.122079 -0.565680
ta 1.057425 -1.335650 0.020717
c 0.129490 -0.289270 -1.731853
h 0.233494 -0.464239 -2.808077
h 2.528041 -0.109094 -0.359933

LM4
E = -455.455292
o -1.613863 2.111073 -0.395164
ta -0.448543 -0.004624 1.491653
c -1.822037 0.911745 0.123873
o -2.599495 0.050963 -0.607584
ta -1.024638 -1.111689 -0.705197
ta 0.309156 1.461843 -0.509686
ta 1.679234 -0.645754 0.001308
c 0.685737 -0.284842 -1.719254
h 0.747639 -0.322763 -2.811313
h 2.166793 1.181387 0.105892

TS4
E = -455.418822
ta -0.423553 -1.116809 -1.030203
c 2.257222 -0.487808 -0.086360
ta 0.651336 -0.719395 1.224329
ta 0.866532 1.362888 -0.313762
ta -1.633091 0.554887 0.444305
o 1.618219 -1.591522 -0.624360
o 2.620742 0.491320 -0.862467
c -0.817798 0.936445 -1.451478
h -1.082401 1.376068 -2.418000
h -2.135237 -1.220996 -0.161190

LM5
E = -455.419221
ta -0.475885 -1.043148 -1.059883
c 2.299605 -0.765326 0.050328
ta 0.524051 -0.691824 1.253899
ta 1.073678 1.245401 -0.304030
ta -1.652560 0.632072 0.396199
o 1.446937 -1.748075 -0.397735
o 2.607206 0.159871 -0.870426

c -0.721898 1.052823 -1.399036
h -0.900643 1.615842 -2.320991
h -2.256405 -1.037782 -0.333009

TS5
E = -455.419311
c 2.256257 -0.864605 0.101139
ta -0.483275 -0.993973 -1.102384
ta 0.471781 -0.674893 1.286081
ta 1.148326 1.193443 -0.298486
ta -1.655000 0.640557 0.385348
o 1.329237 -1.807074 -0.320850
o 2.602224 0.010364 -0.864140
c -0.672810 1.123078 -1.364195
h -0.845607 1.750845 -2.245254
h -2.280573 -0.982806 -0.447277

LM6
E = -455.433694
c 1.999004 0.056343 -1.138867
ta -0.216209 1.374307 -0.660782
ta 0.247054 -1.399599 -0.685237
ta 1.291235 0.002042 1.066168
ta -1.721543 -0.247030 0.487304
o 0.807248 0.137229 -1.955089
o 2.028825 1.237952 -0.433378
c -0.460266 0.945961 1.547956
h -0.657077 1.634125 2.379536
h -2.103083 1.080931 -0.870402

TS6
E = -455.429697
o -2.014763 1.519813 0.286775
ta -1.299407 -0.335523 -1.029038
ta 1.688134 -0.236102 -0.503813
ta 0.132466 1.480945 0.437879
ta -0.116798 -1.253568 0.937344
c -1.897950 0.398180 0.999974
o -0.801592 0.509505 1.949055
c 0.315312 0.647125 -1.724407
h 0.451536 1.282722 -2.608587
h 2.054270 1.341033 0.575356

LM7
E = -455.444405
o -2.372762 1.577335 0.515509
ta -1.119691 -0.748084 -1.016303
ta 1.704181 0.128843 -0.446433
ta -0.255621 1.559360 0.338433
ta 0.171634 -1.263805 0.998242
c -1.973621 0.344757 0.590352
o -1.095257 0.253090 1.738225
c 0.196531 0.655218 -1.702037
h 0.203123 1.196154 -2.654997
h 1.666931 1.789633 0.497625

TS7
E = -455.442421
o -0.914473 0.475570 1.766707
c -2.004653 0.579731 0.472548
o -2.289815 1.791618 0.462763
ta -0.095968 1.571944 0.286599
ta -1.197974 -0.707366 -0.985586
ta 1.701227 -0.050947 -0.477856
ta 0.060690 -1.189496 1.069673
c 0.233749 0.525340 -1.751418
h 1.824907 1.646670 0.387604
h 0.272604 1.023590 -2.726645

LM8
E = -455.534743
o -0.675004 1.804043 1.098419
c -2.125572 -1.104192 0.062431
o -3.028281 -0.326814 -0.320370
ta -1.330298 0.856759 -0.391509
ta -0.218659 -1.617586 0.431615
ta 1.164078 0.182799 -1.218002
ta 0.877863 0.669954 1.237890
c 0.958109 -1.788014 -1.155126
h -0.417259 1.230711 -1.991409
h 1.060497 -2.405894 -2.056331

TS8
E = -455.485808
h -0.308080 0.001479 2.126696
c 0.115489 1.507022 1.619664
ta 1.287845 -0.017044 1.182364
ta -0.795547 1.493596 -0.234269
ta -0.949334 -1.235698 0.272959
ta 0.979951 -0.158133 -1.330489
c -2.362515 0.356847 0.090582
o -2.945477 -0.687770 0.480508
o -0.114890 -1.744368 -1.335216
h 0.100417 2.233748 2.438274

LM9
E = -455.525442
h -0.368745 2.435577 2.003556
c 0.182799 2.126488 1.104075
ta 1.062555 0.226652 1.300869
ta -0.742322 1.394612 -0.607159
ta -0.983684 -1.184436 0.367572
ta 1.155460 -0.443576 -1.134540
c -2.344062 0.396802 -0.027675
o -2.977328 -0.518251 0.547505
o 0.036003 -1.975661 -1.037312
h 0.950328 2.868624 0.804368

TS9
E = -455.474712
h -1.950313 1.134436 0.853660
c -2.394865 -0.202361 -0.321967
ta -0.657787 -1.399430 0.351498
ta -1.052291 1.210497 -0.650159
ta 0.861320 0.501477 1.329979
ta 1.354677 -0.169245 -1.053980
o 0.623447 -1.901693 -0.980876
c -0.464435 2.027855 1.069385

h -0.752397 2.597404 1.960575
o -2.757641 -1.241509 0.275321

LM10
E = -455.499446
h 2.277819 1.777445 -1.376963
c 2.122523 1.207420 -0.448029
ta 1.214757 -0.887789 -0.577750
ta 0.391705 1.474005 0.681819
ta -1.276485 0.114551 -1.195070
ta -0.822993 -0.813850 1.180199
o 0.557619 -2.058976 0.758281
c -0.925299 1.993442 -0.707112
h -1.125028 2.900154 -1.290589
o 2.899136 0.105517 -0.372408

TS10
E = -455.432326
h 2.577107 -0.500558 -0.021679
c 1.938511 0.873865 0.115666
ta -0.090909 1.605319 -0.203139
ta 1.099276 -0.904306 -0.963856
ta -1.521954 -0.682188 -0.360545
ta 0.165376 -0.317457 1.588417
c 1.515148 -1.552368 0.759452
o -1.851306 1.082202 -0.862891
o 1.797671 2.100611 -0.474440
h 2.549400 0.909148 1.025639

LM11
E = -455.501121
h 2.938815 1.158492 -0.859080
c 2.428811 0.170597 -0.926912
ta 1.143964 -0.733890 0.744121
ta 0.511734 1.472955 -0.516187
ta -1.363461 0.360237 0.909509
ta -0.763358 -1.057884 -1.147317
c -1.169562 0.852075 -1.309739
o -0.250277 -0.526685 2.135070
o 2.931703 -0.737935 -0.015571
h 2.446105 -0.201045 -1.956188

TS11
E = -455.466126
h 2.101750 -0.810544 1.138690
c 2.347678 0.124680 -0.293134
ta 1.075071 -1.398871 -0.208835
ta -0.826946 0.006122 1.434528
ta 0.594982 1.487584 -0.112370
ta -1.326695 -0.288369 -1.057556
o -0.568031 1.343418 -1.622629
o 2.695886 1.314945 -0.167341
h -0.433797 2.007129 1.354563
c 0.420859 -1.513925 1.585714

LM12
E = -455.510692
h 2.512859 0.100499 -2.111588
c 2.281762 0.322492 -1.057620
ta 0.695404 1.494992 -0.326438
ta -1.163247 -0.648785 -1.192190
ta 1.102416 -1.093479 0.334580
ta -1.089004 0.313407 1.191190
o 0.098724 -0.968456 1.972714
o 2.898902 -0.548894 -0.210700
h 0.157401 -2.175498 -0.843929
c -1.194735 1.241838 -0.886046

TS12
E = -455.507676
c 1.948122 0.341987 -1.153556
ta -0.835425 -1.023867 -1.160710
ta 0.435517 1.619952 -0.469719
ta 1.192177 -0.898047 0.584534
ta -1.199716 0.321627 1.059398
o 0.046909 -0.631122 2.122574
o 2.811647 -0.158058 -0.215967
h 2.151801 -0.014246 -2.171812
c -1.252390 0.865479 -1.128521
h 0.548972 -2.352711 -0.374236

LM13
E = -455.528423
c -0.226121 1.852836 0.351909
ta -0.362896 0.102761 1.600640
ta 1.469030 0.869830 -0.471945
ta -1.527637 0.300865 -0.707454
ta 0.513899 -1.498702 -0.277676
o -1.071310 -1.410503 -1.390366
o -0.363096 1.868879 -1.128187
h -0.286397 2.864025 0.758366
c 1.579611 -0.279308 1.108157
h -3.104244 0.470763 0.149398

TS13
E = -455.528048
h 2.755248 0.331720 0.488281
ta 0.564394 -0.647385 1.400687
ta 1.420844 0.406071 -0.741982
ta -1.171534 -0.968444 -0.747730
ta -0.958342 1.339726 0.287766
c -1.441323 -0.572328 1.148477
o 0.532640 2.103406 -0.659400
o 0.780513 -1.206552 -1.739582
c 0.798114 -1.790101 -0.376977
h 1.157306 -2.819670 -0.433442

LM14
E = -455.528003
h 1.883632 1.549678 0.818449
ta 0.832414 -0.281171 1.368620
ta 0.985687 1.034332 -0.724230
ta -0.460930 -1.404350 -0.725762
ta -1.560416 0.696225 0.265009
c -0.992685 -1.176444 1.141608
o -0.628080 2.097492 -0.647297
o 1.396150 -0.697315 -1.682573
c 1.709047 -1.177071 -0.337647

h 2.510519 -1.917714 -0.408744

TS14
E = -455.470373
h -1.226873 -0.169676 -1.858015
c -2.233985 -0.209493 -0.663097
ta 0.100885 1.234083 -0.831757
ta -0.355183 -1.391664 -0.792548
ta -1.171558 0.262203 1.160298
ta 1.683727 -0.237761 0.569994
o 2.011025 1.327086 -0.489569
c -0.006155 -1.369139 1.162745
o -2.123485 1.162084 -0.513302
h -3.257191 -0.552705 -0.853981

LM15
E = -455.500407
h -2.958588 -0.624034 -1.443420
c -2.901644 -0.515470 -0.365029
ta -0.525070 1.409035 -0.388228
ta 0.526537 -0.793530 -1.330706
ta -1.128962 -0.786734 0.860185
ta 1.562056 0.087871 0.808571
o 1.076588 1.906080 0.565086
c 0.533097 -1.797801 0.358847
o -2.413628 0.758844 0.062328
h -3.856708 -0.730539 0.124169

LM16
E = -341.010349
ta 1.434113 -0.416233 0.764155
ta -1.436755 -0.404532 0.765476
ta -0.004069 -0.766684 -1.332664
ta 0.006894 1.612073 -0.048176
o 0.003768 1.076147 -1.864916
c -0.007261 -1.734444 0.676261

Pathway from Figure S6
LM1
E = -455.407739
o -5.951890 -0.376457 0.040339
ta 0.704354 -0.501922 1.352496
ta -1.344054 -0.217622 -0.315393
o -3.673415 -0.076765 -0.213170
c -4.827579 -0.231555 -0.084414
ta 0.475455 1.616070 -0.042677
ta 1.560836 -0.695227 -0.926715
c -0.058889 -1.669773 -0.436750
h 2.501049 -0.841311 0.713834
h 1.868996 1.180161 -1.147100

TS1
E = -455.400499
o -4.009795 -0.768478 1.260958
ta -0.023430 -0.780132 1.165840
ta -1.157084 0.046210 -0.997276
o -3.395941 0.129995 -0.794873
c -3.571959 -0.347104 0.287783
ta 0.479616 1.594842 0.392552
ta 1.747047 -0.635972 -0.565207
c 0.055256 -1.474382 -1.000191
h 1.798816 -1.596250 1.024683
h 2.178436 1.211866 -0.180279

LM2
E = -455.454634
o -2.346301 -1.540841 0.939416
ta -0.295024 -1.442327 0.292210
ta -1.145937 0.839727 -0.868599
o -3.070261 0.527675 0.150839
c -2.331206 -0.476127 0.267207
ta 0.468588 0.923735 1.242672
ta 1.705361 -0.119300 -0.678242
c 0.027300 -0.544695 -1.648020
h 1.576461 -1.915659 -0.004064
h 2.071343 1.411991 0.439965

TS2
E = -455.449509
c 2.085530 0.652814 0.187672
ta -0.074048 -0.745325 1.344359
ta 1.151757 -0.814092 -0.940934
ta 0.038661 1.545922 0.064766
ta -1.740244 -0.236053 -0.484152
o 2.690013 -0.394385 0.602548
o 2.105876 1.876331 0.522812
c -0.265326 0.360460 -1.686523
h -1.914639 1.602779 0.022706
h -1.830843 -1.320892 1.132671

LM3
E = -455.469932
c 1.866407 0.490970 -0.507675
ta 0.726556 -0.124497 1.362286
ta 0.671029 -1.228743 -0.945377
ta -0.186414 1.508911 -0.462392
ta -1.726963 -0.420132 0.234257
o 2.561465 0.341520 0.621969
o 1.739559 1.689653 -1.058762
c -0.809645 -0.091365 -1.588595
h -2.033810 1.433715 0.322443
h -1.062156 -0.774996 1.969005

TS3
E = -455.447387
o 2.413092 -0.423599 0.612336
ta 1.158893 -0.264527 -1.188787
ta -0.165176 1.531343 0.398177
ta -1.698380 -0.318908 -0.396305
ta 0.206727 -1.191082 1.006283
c 1.577322 0.511608 1.003308
o 1.763055 1.752928 1.279040
c -0.473914 0.736284 -1.596379
h -2.074597 1.300843 0.541475
h -1.605621 -1.671163 1.072036

LM4
E = -455.447723

o -2.495583 -0.220852 -0.469117
ta -1.181668 -0.023331 1.179231
ta 0.259217 1.487166 -0.525399
ta 1.691593 -0.359995 0.415050
ta -0.301232 -1.341384 -0.846978
c -1.518735 0.350250 -1.175428
o -1.517065 1.569105 -1.606400
c 0.566836 0.854319 1.522582
h 2.153690 1.125250 -0.683196
h 1.501432 -1.798046 -0.994498

TS4
E = -455.446135
ta -0.341930 -1.232942 -1.004687
o -2.643540 0.120553 -0.194987
ta -1.176238 -0.116232 1.196588
ta 0.325226 1.472146 -0.490254
ta 1.682030 -0.421808 0.459444
c -1.643108 0.445920 -1.035105
o -1.475935 1.695276 -1.409637
c 0.565686 0.825152 1.527329
h 2.217579 1.051235 -0.628214
h 1.499329 -1.389207 -1.247802

LM5
E = -455.450943
ta -0.338576 -0.152495 -1.518719
o -2.549752 0.378147 0.468135
ta -1.198740 -0.955630 0.765101
ta 0.418972 1.492300 0.430755
ta 1.607838 -0.767437 0.180253
c -1.695851 1.084280 -0.384906
o -1.399248 2.295126 0.048098
c 0.361436 -0.087060 1.694229
h 2.288679 0.992195 0.091386
h 1.576737 -0.383608 -1.666643

TS5
E = -455.441027
ta 0.023858 -1.367592 -0.846155
ta -1.108437 -0.235397 1.199756
ta 1.813251 0.037160 0.404189
ta -0.179722 1.430627 -0.593999
o -1.756053 0.965294 -1.659256
o -2.714426 -0.266782 -0.022888
c -1.831180 -0.237086 -1.021111
c 0.498828 0.980047 1.468339
h 1.922708 -1.711829 -0.484700
h 1.761924 1.535770 -0.698211

LM6
E = -455.442403
ta 0.180667 -1.424942 -0.808793
ta -1.153007 -0.343418 1.105494
ta 1.731291 0.121917 0.424748
ta -0.243626 1.536003 -0.518137
o -1.524219 0.863162 -1.812694
o -2.639354 -0.353536 -0.240144
c -1.644193 -0.335992 -1.144973
c 0.339733 1.013177 1.481358
h 1.868131 -1.795083 0.234621
h 1.648515 1.717085 -0.672041

TS6
E = -455.438282
o -0.852180 0.096934 2.022562
c -1.856321 0.583975 0.897258
ta -0.094709 1.547682 0.465852
ta -1.218344 -0.264979 -1.122485
ta 1.683053 0.017925 -0.531090
ta 0.094729 -1.243768 0.938702
c 0.440327 -1.285440 -1.285945
o -2.762485 -0.173705 0.516571
h 1.467674 1.996599 -0.623715
h 2.020412 -1.324508 0.821262

LM7
E = -455.515235
o 0.020938 1.457363 1.634675
c -2.503402 0.495466 -0.236724
ta -0.729143 1.438666 -0.149212
ta -1.100601 -1.208536 -0.179243
ta 1.438484 -0.033213 -1.103377
ta 0.838276 -0.234183 1.350013
c 0.838384 -1.671067 -0.327073
o -3.262655 -0.454793 -0.334083
h 0.664533 1.822555 -1.406770
h 2.627184 -0.069127 0.357596

TS7
E = -455.515779
ta -0.703272 1.445768 -0.129219
ta -1.119938 -1.197826 -0.154493
ta 1.417601 -0.045499 -1.126198
ta 0.847444 -0.246283 1.343143
c 0.802584 -1.682420 -0.355975
c -2.489345 0.537422 -0.294762
o 0.077492 1.460573 1.647768
o -3.250253 -0.411397 -0.406829
h 0.626063 1.819216 -1.459467
h 2.622637 -0.142329 0.310307

LM8
E = -455.523303
ta -0.608947 1.452962 0.270446
ta -1.197056 -1.126650 -0.204656
ta 1.227842 0.202887 -1.240093
ta 1.005113 -0.579669 1.152131
c 0.529544 -1.569881 -0.991609
c -2.403090 0.736699 -0.365400
o 0.390867 1.041553 1.911058
o -3.231321 -0.140843 -0.509650
h 0.213190 1.949863 -1.364639
h 2.584117 -0.472176 -0.085990

TS8
E = -455.519520
ta 0.519173 1.378777 -0.615281

ta 1.240333 -1.119029 0.177958
ta -0.934510 0.474928 1.318856
ta -1.226481 -0.785657 -0.851456
c -0.272807 -1.357508 1.341239
c 2.332248 0.804983 0.073290
o -0.772737 0.624167 -1.969538
o 3.187794 0.020311 0.431607
h 0.192701 2.104185 1.119050
h -2.561354 -0.223294 0.501560

LM9
E = -455.519867
ta 0.382965 1.308297 -0.807135
ta 1.292200 -1.071924 0.155382
ta -0.796412 0.603725 1.338091
ta -1.251718 -0.908209 -0.661706
c -0.118156 -1.249696 1.446029
c 2.213691 0.935981 0.001015
o -0.952981 0.350886 -1.990873
o 3.069955 0.226731 0.501929
h 0.189032 2.240174 0.857424
h -2.471525 -0.006757 0.573752

TS9
E = -455.487205
h 1.999455 1.139897 1.502370
ta -0.822578 -0.243716 1.447707
ta 1.239209 -1.055866 -0.373628
ta 0.511217 1.510925 -0.043893
ta -1.305915 -0.267495 -1.007251
c -0.478370 -1.822456 0.291492
c 2.325364 0.732672 0.424519
o 3.052501 -0.324996 0.385963
o -0.922124 1.539843 -1.390752
h -2.525610 -0.220925 0.565613

LM10
E = -455.500444
h 3.082208 1.048110 1.389777
ta -1.432925 0.108599 1.092185
ta 1.133204 -1.173662 0.322387
ta 0.655340 1.490785 -0.027157
ta -0.821532 -0.442845 -1.301027
c -0.814599 -1.632488 0.636024
c 2.491296 0.661018 0.538421
o 2.979142 -0.445082 -0.002251
o -0.049022 1.226687 -1.793022
h -2.571674 -0.222125 -0.380602

TS10
E = -455.491023
ta 1.319283 -0.393516 -1.091941
ta -1.121861 -1.233436 0.052643
ta -0.626967 1.434825 -0.474209
ta 0.862960 0.118413 1.354622
c 0.909653 -1.682604 0.314586
c -2.436643 0.462383 -0.413974
o -2.894913 -0.420852 0.493120
o 0.144508 1.914100 1.173153
h 2.517038 0.243692 0.242029
h -2.991074 0.512779 -1.377286

LM11
E = -455.493005
ta 1.031219 -0.826216 -1.065206
ta -1.168944 -1.110306 0.388279
ta -0.588906 1.349134 -0.718977
ta 1.110510 0.421840 1.173091
c 0.846238 -1.562140 0.778664
c -2.404579 0.594031 -0.093956
o -2.726305 -0.033379 1.050080
o 0.477232 2.175589 0.594574
h 2.481623 0.196968 -0.093325
h -3.162178 0.559054 -0.906791

TS11
E = -455.464623
h -1.704771 0.766159 1.137676
ta 0.583115 1.342783 0.593983
ta -1.659237 0.272003 -0.848827
ta 1.337234 -0.942323 -0.567740
ta -0.685885 -0.898668 1.114432
c -0.588757 -1.356008 -1.065575
o -0.488340 1.759303 -0.934123
c 2.561296 0.886488 0.063665
o 2.664325 0.470661 -1.203199
h 3.470137 0.724189 0.667466

LM12
E = -455.515524
h -0.370955 2.279806 0.962227
ta 0.748856 1.383972 -0.252689
ta -0.854198 -0.627080 -1.255071
ta 0.983050 -1.115212 0.545356
ta -1.364980 0.375935 1.066517
c -1.054783 -1.492522 0.726676
o 0.025010 0.916761 -1.967193
c 2.601496 0.510710 0.312660
o 2.889613 -0.749564 -0.004383
h 3.344566 0.987640 0.974020

TS12
E = -455.463895
h 1.469579 0.616473 1.681916
c 2.471290 0.553665 0.508011
o 2.905500 -0.659104 0.221887
ta 1.235802 -0.679395 -0.925011
ta -0.208671 -0.777465 1.260009
ta 0.458636 1.490468 0.199120
ta -1.887579 -0.094689 -0.493868
c -0.604583 -1.517077 -0.805517
o -1.225943 1.708628 -0.693863
h 3.226034 1.226708 0.940730

LM13
E = -455.503908
h 2.754408 1.119591 1.030762
c 2.667597 0.627503 0.020552

```
o 2.935100 -0.740964 0.121927
ta 1.105693 -1.027133 -0.510387
ta -0.785270 -0.445066 1.433181
ta 0.564210 1.446534 0.216536
ta -1.366684 -0.023039 -1.040967
c -0.674901 -1.806761 0.029434
o -0.791064 1.787735 -1.099700
h 3.326928 1.137182 -0.688961
```

References

[1] D. Neuwirth, J. F. Eckhard, K. Lange, B. Visser, M. Wiedemann, R. Schröter, M. Tschurl, U. Heiz, *Int J Mas Spectrom* **2015**, *387*, 8-15.

[2] U. Heiz, F. Vanolli, L. Trento, W. D. Schneider, *Rev Sci Instrum* **1997**, *68*, 1986-1994.

[3] T. Masubuchi, J. F. Eckhard, K. Lange, B. Visser, M. Tschurl, U. Heiz, *Rev Sci Instrum* **2018**, *89*, 023104.

[4] M. Sakurai, K. Watanabe, K. Sumiyama, K. Suzuki, *J Chem Phys* **1999**, *111*, 235-238.
